# Polyglutamine Ataxias: Our Current Molecular Understanding and What the Future Holds for Antisense Therapies

**DOI:** 10.3390/biomedicines9111499

**Published:** 2021-10-20

**Authors:** Craig S. McIntosh, Dunhui Li, Steve D. Wilton, May T. Aung-Htut

**Affiliations:** 1 Molecular Therapy Laboratory, Centre for Molecular Medicine and Innovative Therapeutics, Health Futures Institute Murdoch University, Discovery Way, Murdoch, WA 6150, Australia; c.mcintosh@murdoch.edu.au (C.S.M.); dunhui.li@murdoch.edu.au (D.L.); s.wilton@murdoch.edu.au (S.D.W.); 2 Perron Institute for Neurological and Translational Science, Centre for Neuromuscular and Neurological Disorders, The University of Western Australia, Nedlands, WA 6009, Australia

**Keywords:** polyglutamine, ataxia, antisense oligonucleotides, expansion diseases

## Abstract

Polyglutamine (polyQ) ataxias are a heterogenous group of neurological disorders all caused by an expanded CAG trinucleotide repeat located in the coding region of each unique causative gene. To date, polyQ ataxias encompass six disorders: spinocerebellar ataxia types 1, 2, 3, 6, 7, and 17 and account for a larger group of disorders simply known as polyglutamine disorders, which also includes Huntington’s disease. These diseases are typically characterised by progressive ataxia, speech and swallowing difficulties, lack of coordination and gait, and are unfortunately fatal in nature, with the exception of SCA6. All the polyQ spinocerebellar ataxias have a hallmark feature of neuronal aggregations and share many common pathogenic mechanisms, such as mitochondrial dysfunction, impaired proteasomal function, and autophagy impairment. Currently, therapeutic options are limited, with no available treatments that slow or halt disease progression. Here, we discuss the common molecular and clinical presentations of polyQ spinocerebellar ataxias. We will also discuss the promising antisense oligonucleotide therapeutics being developed as treatments for these devastating diseases. With recent advancements and therapeutic approvals of various antisense therapies, it is envisioned that some of the studies reviewed may progress into clinical trials and beyond.

## 1. Introduction

The polyglutamine (polyQ) spinocerebellar ataxias (SCAs) are a heterogenous group of neurodegenerative diseases, all arising from a CAG (glutamine) expansion in their respective genes [1,2,3,4,5,6]. There are currently six described polyQ SCAs, SCA1, SCA2, SCA3, SCA6, SCA7, and SCA17, with the number denoting the timeline in which the diseases were first discovered (Table 1)—SCA1 was the first described SCA disease, SCA2 the second, etc. The polyQ SCAs are all caused by a variable CAG expansion located within the coding region of their respective genes; this expansion directly causes the inclusion of an extended polyQ tract in the encoded proteins, leading to conformational changes giving the proteins a toxic gain of function [7]. Although the presence of unexpanded polyQ tracts does not directly lead to phenotypic modification, there appears to be a variable threshold in each gene, where if reached causes disease. Due to the nature of this type of mutation, there appears to be a common theme—the larger the expansion is, the earlier is the age of onset, and the more severe is the disease phenotype [8]. Additionally, these diseases typically follow what is known as ‘genetic anticipation’, whereby the expansion can increase in size with each successive generation [8,9]. Although caused by the same expansion mutation, the diseases present as clinically heterogenous, with some common features such as ataxia, speech and swallowing difficulties, as well as impaired hand, gait, and motor functions [10]. Spinocerebellar ataxia type 3 is the most common of the SCAs, with SCA17 being one of the rarest. It should be noted that the frequency distribution of the SCAs can vary quite widely between various ethnic populations. A prime example of this is the relatively high frequency of SCA6 observed in Taiwanese and Japanese populations when compared with their Caucasian counterparts [11].

The six polyQ SCAs form a larger group of diseases known as the polyQ diseases, which include Huntington’s disease (HD), spinal and bulbar muscular atrophy (SBMA), and dentatorubral–pallidoluysian atrophy (DRPLA) [7,12]. Currently, there are little to no effective treatment strategies for any of the polyQ SCAs or other polyQ diseases. With that said, there has been significant advancement of pre-clinical and clinical studies in recent years that is delivering some hope for the patients suffering from these progressive diseases [13]. This review will focus on our genetic understanding of the polyQ SCAs (termed SCAs from here on) and the prospect of new and novel therapies for a class of progressive neurodegenerative diseases.

## 2. The CAG Expansion

**Figure 1:**
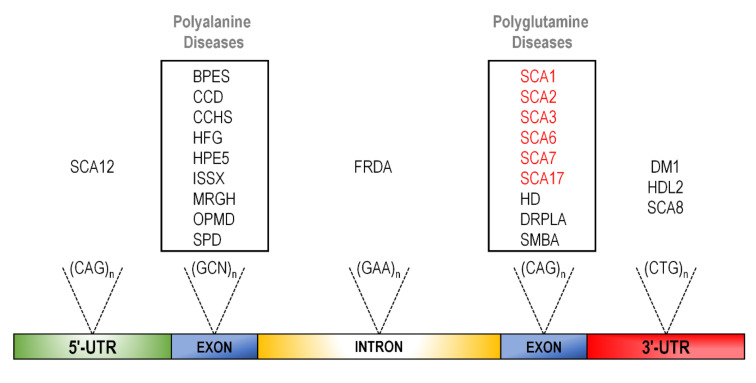
Relative pre-mRNA locations of tri-nucleotide expansions causing human disease. Type of expanded trinucleotides found in respective locations are shown above the transcript: SCA12 = spinocerebellar ataxia type 12; BPES = blepharophimosis, ptosis and epicanthus inversus; CCD = cleidocranial dysplasia; CCHS = congenital central hypoventilation; HFG = hand–foot–genital syndrome; HPE5 = holoprosencephaly 5; ISSX = X-linked infantile syndrome; MRGH = mental retardation with isolated growth hormone deficiency; OPMD = oculopharyngeal muscular dystrophy; SPD = synpolydactyly. Intron: GAA repeat: FRDA = Friedreich’s ataxia; SCA1, 2, 3, 6, 7, 17 = spinocerebellar ataxia type 1, 2, 3, 6, 7, 17; HD = Huntington’s disease; DRPLA = dentatorubral–pallidoluysian atrophy; SMBA = spinal and bulbar muscular atrophy;DM1 = myotonic dystrophy type 1; HDL2 = Huntington’s-disease-like 2; SCA8 = spinocerebellar ataxia type 8. Polyglutamine ataxias are in red.

Expansion disorders caused by unstable, expanded microsatellites currently account for over 40 genetically distinct diseases, the majority of which present as neurological pathologies [14,15,16,17,18]. Of these, tri-nucleotide expansion diseases are the most common, and more specifically, CAG expansions located in coding regions are the most prevalent of all [19] (Figure 1).

With dozens of diseases arising from trinucleotide expansions at various locations within the genome, these repetitive sequences are highly predisposed to expansion due to the unusual secondary structure they appear to generate [20]. These secondary structures appear to hinder normal cellular replication, recombination, and repair of DNA, leading to the insertion of a variable number of trinucleotide repeat sequences. Of these secondary structures, slip-strand mispairing appears to be the most common mechanism in polyQ expansion diseases [20,21,22].

## 3. Common Pathological Features

**Figure 2:**
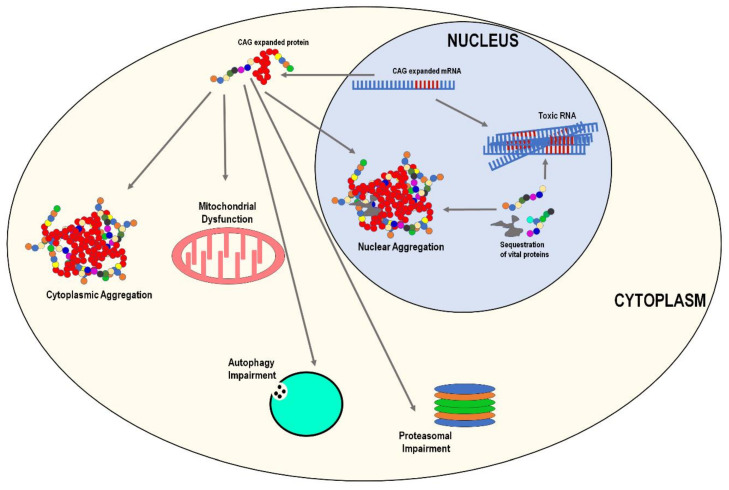
Schematic representation of some of the common cellular pathologies associated with polyglutamine spinocerebellar ataxias (SCAs). Polyglutamine (polyQ) expanded mRNA has the ability to form toxic RNA compounds prior to being translated to a polyQ expanded protein. Following translation, polyQ expanded proteins form neuronal cytoplasmic and nuclear inclusions. The expanded polyQ protein causes both proteasomal and autophagy impairment. The polyQ expanded proteins also have the ability to cause mitochondrial dysfunction. PolyQ amino acids are presented as red circles, while CAG nucleotides on the polyQ mRNA as red ‘fingers’ on the mRNA transcript.

Although clinically heterogenous, there are several common pathological features shared by the six SCAs. The most notable being neuronal nuclear/cytoplasmic aggregation of polyQ proteins and various vital sequestered proteins (Figure 2). Apart from neuronal aggregation, other common pathological features include mitochondrial dysfunction and oxidative stress, autophagy impairment, proteasomal impairment, neuroinflammation, and potential toxic RNA (Figure 2). We will briefly describe the overall pathogenic mechanisms; however, disease-specific details will be discussed in later sections.

### 3.1. Protein Aggregation

The misfolding and aggregation of mutant proteins in various neuronal cells is a hallmark feature of SCA diseases, although the exact role protein aggregation plays in pathogenesis remains unclear, in relation to the idea of cause versus effect. However, there is a consensus among scientists that protein aggregates play a toxic role, as they have been reported to sequester non-expanded proteins and contribute to the proteostatic collapse seen in SCAs [23,24,25,26]. Conversely, protein aggregation has been intensively studied in a host of neurodegenerative diseases, not limited to SCAs, and there is still no concrete evidence that answers the question ‘Are protein aggregates the cause of neurodegeneration, a consequence/effect of the given neurodegenerative disease, or an epiphenomenon of neurodegenerative diseases [23,27,28,29,30,31]?’ Although this is a complex question, the generation of transgenic models expressing polyQ expansions with phenotypic symptoms that reflect the human condition, suggests protein aggregation could not simply be an epiphenomenon event [32,33,34,35,36]. What is more plausible is a combination of causation and consequence [37,38], in that aggregation could be a consequence of the conformational changes observed in expansion, and the subsequent sequestration of other non-expanded proteins may contribute to disease pathogenesis.

Despite having similar clinical pathology, the causative proteins themselves share no homology in amino acid sequence, secondary or tertiary structures, and no common biological function. Therefore, an expanded polyQ stretch is the only characteristic shared among these proteins, suggesting the integral role of the polyQ tract in disease. Although the polyQ tract itself appears to be sufficient for disease, the conformational changes and misfolding of the associated disease proteins are significant factors in aggregation and can promote the formation of β-sheets. Aggregates exist as a heterogenous group of proteins, where misfolded, unfolded, and intermediately folded proteins combine. In cells where no expansions exist, misfolded proteins are either refolded correctly or degraded; however, when an expansion occurs in a given gene, it is thought that molecular chaperones and proteasomes are simply overwhelmed with patron proteins, ultimately leading to insoluble aggregation.

### 3.2. Mitochondrial Dysfunction and Oxidative Stress

In the last 20 years, it has become increasingly clear that mitochondrial dysfunction may impact neuronal function in many of the SCAs. Neuronal tissue, similar to many other tissues, requires functional mitochondria for most cellular processes, including cell proliferation, differentiation, and apoptotic cell death [39]. There is a range of evidence supporting the imbalance of the oxidative–antioxidant system, leading to oxidative stress and aberrant mitochondrial morphology in patients and various models of SCA1, 2, 3, and 7 [40,41,42,43,44,45,46]. One compound of interest is coenzyme Q10 (CoQ10), a vitamin-like molecule, that plays a particularly important role in protecting mitochondria from free-radical oxidative damage [47]. Therefore, supplementation with CoQ10 could be a highly attractive therapy for SCAs where oxidative stress plays a significant role, e.g., SCA2. In fact, several studies have reported some symptomatic relief in patients who were supplemented with CoQ10, at a range of 300–3000 mg/day, without any significant adverse effects [48,49].

The role of oxidative stress in SCA disease progression was highlighted for the first time by a 2018 study that demonstrated elevated levels of circulating markers of oxidative stress in patients diagnosed with SCA7, which appeared to correlate with disease progression and severity [50]. The study sought to determine peripheral levels of various oxidative stress markers, such as lipid and protein damage markers, as well as antioxidant defence markers in 29 SCA7 patients, compared with 28 healthy individuals. Results showed symptomatic SCA7 patients exhibit damage to various lipids and proteins and enhanced activity of some antioxidant enzymes [50]. The group concluded that biomarkers of oxidative stress could be a useful tool in studying the molecular disease progression of SCA7. Additionally, SCA3 patients have also been shown to have decreased peripheral antioxidant capacity and increased reactive oxygen species, with GSH-Px being identified as the most promising biomarker of disease severity [51]. Interestingly, significant oxidative stress was only present in SCA3 patients following disease onset, an observation also seen in SCA7 patients [50,51]. This may be in part due to exhaustion in antioxidant defence mechanisms which could play a role in the progression of the SCA3 phenotype.

### 3.3. Proteasomal and Autophagy Impairment 

Macro-autophagy (autophagy) and the ubiquitin–proteasome system (UPS) are two similar intracellular protein degradation systems. In non-neuronal cells, autophagy is typically dedicated to recovery from nutrient stress, while in neurons, the focus has been adapted to clearing dysfunctional cellular components and degrading misfolded proteins [52]. Therefore, it stands to reason that these two major neuronal systems devoted to clearing aberrant proteins may be highly dysregulated in SCA diseases.

Autophagy impairment is best characterised in SCA3, where the brains of SCA3 patients show an alteration in autophagy machinery, increased accumulation of autophagosomes, and decreased levels of beclin-1 [53,54], with beclin-1 being a critical protein involved in autophagy by inhibiting apoptosis and promoting cell survival during stress [55]. Moreover, the presence of an expanded polyQ tract in ATXN3 competes and impairs the initiation of autophagy [56,57]. Interestingly, unlike other SCAs (SCA1 and SCA7), SCA3 disease pathogenesis was initially thought not to affect the proteasome. However, overexpression of polyQ expanded ATXN3 appeared to perturb the function of the UPS and subsequently led to an abnormal increase in proteasome substrates. It is thought that the polyQ tract may affect the key chaperones by sequestering them into aggregates, indirectly causing a nuclear UPS malfunction [58]. Intriguingly, a 2019 study assessed the potential of a traditional Chinese herbal medicine NH037 (from *Pueraria lobata*) and its constituent, daidzein, as UPS enhancing therapeutics on neurons derived from a SCA3 patient iPSC line [59]. Following proteasome inhibitor (MG132) treatment, NH037 and daidzein treatments appeared to rescue some of the proteasome activity, as well as reducing oxidative stress and caspase 3 activity [59]. Although promising, this treatment would have to be validated in in vivo models for its effect in a heterogenous cellular population. The same group has also shown in vitro benefits of other traditional herbal medicines for various SCAs, including SCA17 [60].

### 3.4. RNA Toxicity

The RNA toxicity due to CAG repeat expansion could be through several mechanisms such as aberrant alternative splicing [61], RAN-initiated translation [62], bidirectional transcription [63], potential involvement in RNAi pathways [64]. Additionally, a growing body of evidence shows a role for the secondary structure of the CAG repeat causing toxicity. Similar to the CUG repeat, CAG repeats tend to fold into stable hairpin structures when the repeat length is long enough, and these structures can be predisposed to form RNA foci [64]. The repeat length appears to be crucial for the stability of the hairpin, as shorter repeats tend to form semi-stable structures, while large expanded CAG/CUG repeats are more stable [65].

Of the SCA diseases, the role of RNA toxicity in pathogenesis has best been described in SCA3. Interestingly, the CAG codon itself appears to play a major role in RNA toxicity, as *Drosophila* expressing a pure CAG repeat displayed progressive neuronal dysfunction [66]. Conversely, *Drosophila* expressing an interrupted CAA/CAG repeat dramatically mitigated the toxicity of the RNA, indicating the importance of CAG containing repeats in toxicity [66]. However, RNA toxicity may not play a role in SCA17; a 2020 study found that CAG-expanded repeat RNA alone is not sufficient enough to account for neuronal apoptosis in vitro [67]. Rather, the polyQ-containing protein is responsible for the neuroinflammation observed [67].

### 3.5. Neuroinflammation

Neuroinflammation is a common feature in many neurodegenerative diseases, such as motor neuron disease, HD, and SCA3 [68]. Neuroinflammation often occurs as a byproduct of oxidative stress, with the inflammation itself also reducing cellular antioxidant capacity, creating a vicious pathogenic cycle [69]. This is supported by the fact that various inflammatory genes have been shown to be upregulated in ATXN3 expanded cells and the brains of SCA3 patients [70]. The upregulation of MMP-2 and amyloid β-protein was most apparent in the pontine neurons that contained nuclear inclusions [70]. Recently, Chen et al. demonstrated significant anti-inflammatory effects of various small molecule compounds, demonstrating their effect on reducing inflammatory markers such as NO, IL-1β, TNF-α, and IL-6, in IFN-γ-induced ATXN3/Q75-GFP SH-SY5Y and human HMC3 microglia [68]. Although highly preliminary, the study demonstrates a potential therapeutic avenue for neuroinflammation-associated diseases.

## 4. Spinocerebellar Ataxia Type 1 (SCA1)

**Figure 3:**
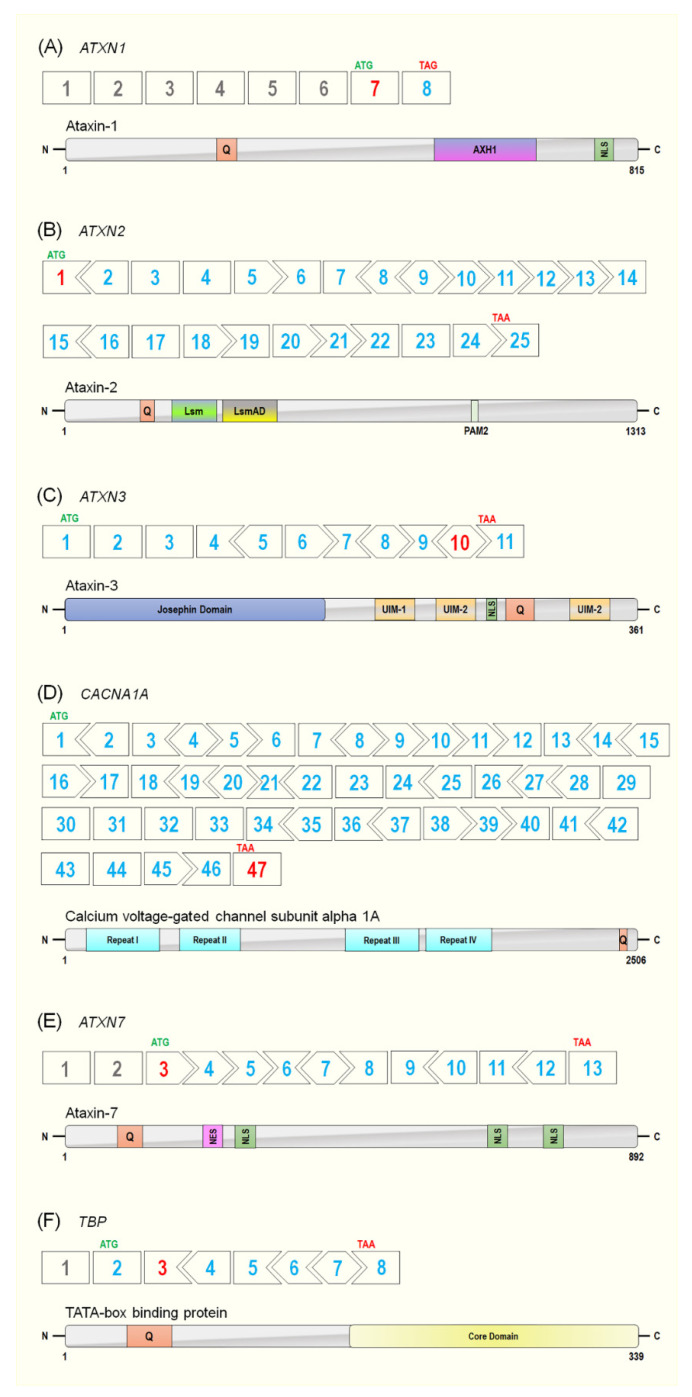
Schematic representation of the polyglutamine spinocerebellar ataxia causative gene reading frames and their respective protein organisations: (**A**) *ATXN1* reading frame and the encoded Ataxin-1 protein organisation; (**B**) *ATXN2* reading frame and the encoded Ataxin-2 protein organisation; (**C**) *ATXN3* reading frame and the encoded Ataxin-3 protein organisation; (**D**) *CACNA1A* reading frame and the encoded Calcium voltage-gated channel subunit alpha 1A protein organisation; (**E**) *ATXN7* reading frame and the encoded Ataxin-7 protein organisation; (**F**) *TBP* reading frame and the encoded TATA-box binding protein organisation. In-frame exons are represented as rectangles, whereas those bounded by partial codons are represented with chevron sides. Grey exons represent non-coding exons, while blue exons represent coding exons, and red exons represent the exon in which the polyQ repeat is located: AXH1 = AXH1 domain; Lsm = like-Sm domain; LsmAD = Lsm associated domain; NES = nuclear export signal; NLS = nuclear localisation signal; PAM2 = PABP-interacting motif 2; UIM = ubiquitin-interacting motif. Q = polyglutamine tract.

Spinocerebellar ataxia type 1 was the first of the SCAs to be described in 1993, hence the designation of type 1. The causative gene for SCA1 is *ATXN1*, with the major transcript (ENST00000436367.6) containing 8 exons, although only the last 2 exons encode the 815 amino acid (aa), 87 KDa protein, ataxin-1 (ATXN1) (Figure 3). The polyQ tract is located within exon 7, the first coding exon. Healthy individuals have a repeat range of 6–35, and incomplete penetrance is associated with 36–40 repeats, while the pathogenic range is 41–89 (Table 1). The ATXN1 protein has several roles within transcriptional regulation and RNA metabolism. In fact, ATXN1 is able to act as a transcriptional repressor [71,72]; that is, ATXN1 and the related ATXNL1 (ATXN1-like) protein have both been shown to compromise the Notch signalling pathway [71]. Interestingly, ATXNL1, a highly conserved paralog of ATXN1, has been implicated as a targeted therapeutic approach for SCA1. With initial work in *Drosophila melanogaster* showing that overexpression of ATXN1L suppressed ATXN1 associated neurotoxicity [73], Zoghbi et al. used a SCA1 knock-in mouse model to generate a targeted *Atxn1l* duplication [74]. The group found that mice with elevated *Atxn1l* levels had reduced neuropathology. It is believed that overexpression of *Atxn1l* displaces *Atxn1* from its native complex with Capicua. A subsequent study appears to confirm this potential therapeutic avenue of ATXN1L, whereby overexpression of *Atxn1l* via gene therapy viral vectors resulted in SCA1 transgenic mice displaying improved histological phenotype and behaviour [75].

Spinocerebellar ataxia type 1 presents phenotypically as typical, progressive cerebellar ataxia with problems in gait and balance, with associated disturbances in oculomotor movements. Additionally, patients may often present with bulbar and pyramidal symptoms, and as the disease progresses, the cognitive decline becomes increasingly evident with impaired mood and judgment being most commonly reported. 

The symptoms of SCA1 typically present in the 3rd to 4th decade, with an age of onset ranging from juvenile to over 60 years of age. As previously stated, the earlier the age of onset is, the more severe is the decline, which is inversely proportional to the polyQ length (true for all SCAs). Notably, SCA1 has been described as one of the fastest progressing SCAs. This may be due to the functional role of ATXN1 being severely compromised by an expanded polyQ repeat, but further evidence is required to make a definitive statement. Pathologically, SCA1-affected individuals present with brain stem and cerebellar degeneration, with profound loss of Purkinje cells in the cerebellum [76,77]. Additionally, SCA1 patients have also been found to have atrophy of the middle cerebellar peduncles and ventral pons [77].

## 5. Spinocerebellar Ataxia Type 2 (SCA2)

Spinocerebellar ataxia type 2 is thought to be the second most prevalent SCA after SCA3, with estimates that SCA2 accounts for 13% of all autosomal dominant cerebellar ataxia cases [78]. A founder effect has been reported in Holguin, Cuba [79,80], where the prevalence in this north-eastern region is almost sevenfold higher than the national average [79,80]. Countries in the Americas are known to have large founder effects of numerous SCAs–SCA2 (north-eastern Cuba); SCA3 (southern Brazil) and SCA7 (south-eastern regions of Mexico) [81].

The causative gene for SCA2 is *ATXN2,* which spans approximately 130 kb of genomic DNA and consists of 25 exons (ENST00000550104.5) (Figure 3), with the pathogenic CAG repeat located in the first exon. Healthy individuals have a repeat range of 17–29, and incomplete penetrance is observed with 30–36 repeats, while SCA2 disease onset occurs at a range of 37–100 or more repeats (Table 1). The *ATXN2* gene encodes a 1313 aa, 124 kDa protein, ataxin-2 (ATXN2) (Figure 3). The normal function of ATXN2 has not been fully described, although it is believed to be involved in various RNA-processing and metabolism pathways. More recently, studies have shown that ATXN2 may play roles in cellular metabolism, stress responses, circadian rhythms as well as mediating cytoplasmic polyadenylation [82,83,84,85,86]. Interestingly, the intermediate, non-penetrative expansion size in ATXN2 has been associated with other diseases, specifically amyotrophic lateral sclerosis (ALS) and frontotemporal dementia (FTD) [87]. Extensive studies have shown that ATXN2 is a strong modifier of TAR DNA-binding protein 43 (TDP-43) toxicity—a defining protein implicated in ALS. In both *Drosophila* and murine models, overexpression of *ataxin-2* enhances TDP-43 toxicity, while conversely, reduces *Atxn2*-attenuated TDP-43 toxicity [88,89]. In fact, a 2017 study by Becker et al. showed that therapeutic reduction in *Atxn2* reduced mortality and pathology in a TDP-43 mouse model (discussed in more detail in later sections) [89].

Spinocerebellar ataxia type 2 is distinct from other SCAs by virtue of an exceedingly slow saccade phenotype. While other common symptoms include ataxic movements and myoclonus, they can sometimes present with typical Parkinson-like, ALS, or FTD phenotypes [90,91,92]. In regard to neurodegeneration, the areas typically affected include the cerebellum and brainstem, with severe degeneration of cerebellar Purkinje cells and granule cells combined with neuron loss and gliosis of the pons [93,94].

## 6. Spinocerebellar Ataxia Type 3 (SCA3)

Spinocerebellar ataxia type 3 is the most common subtype of autosomal dominant ataxia worldwide, with large founder regions across the globe, including Brazil, Portugal, and Japan [95]. The highest frequency of SCA3 is observed in the islands of Azores (West of Lisbon), where the incidence of SCA3 is its highest on the island of Flores (1:140) [96]. SCA3 is also known as Machado–Joseph disease as it was first described in 1972 in a group of Portuguese immigrants known to be decedents of William Machado, living in Massachusetts [97].

The causative gene for SCA3 is *ATXN3*, with the CAG repeat located in the penultimate exon (exon 10) of the major transcript (ENST00000644486.2). Healthy individuals have a repeat range of 7–44 CAG repeats, while non-penetrative individuals possess 45–54 repeats, and diseased individuals harbour 55–89 repeats (Table 1) [98]. The *ATXN3* gene spans a genomic region of 48 kb and consists of 11 exons—encoding a major 361 aa ATXN3 protein isoform (Figure 3) [98]. Ataxin-3 is a ubiquitously expressed protein involved in numerous cellular pathways, acting as an isopeptidase, and involved in deubiquitination, proteasomal protein degradation, and regulation of misfolded proteins [75]. Located at the N-terminal of ATXN3 is the main functional domain of the protein—the Josephin domain—containing the amino acids (cysteine 14, histidine 119, and asparagine 134), crucial for its isopeptidase activity. Additionally, there are two nuclear export signals (NES), and the C-terminal contains three ubiquitin-interacting motifs (UIMs), the polyQ tract, and a nuclear localisation signal [99]. It should be noted, however, that up to 20 potential protein isoforms have been reported, and 56 splice variants isolated in blood [100,101]. A study by Harris et al. in 2010 demonstrated that there are two main splice isoforms (one containing 3UIMs and the other containing 2UIMs) through alternative splicing of the last exon [99]. The isoform described above, with 3 UIMs, is the predominant isoform in the brain, and while the two isoforms display similar deubiquitinating actives, they have different aggregation propensities. This group demonstrated that the 2UIM protein isoform is more prone to aggregation while being degraded more rapidly by the proteasome, highlighting that specific isoforms could contribute to selective neurotoxicity [99,102].

The typical age of onset for SCA3 is in the fourth decade, with an average life span of 10 years following diagnosis. Aside from the common features mentioned above, SCA3 can be distinguished from other SCAs by the presence of pyramidal and extrapyramidal motor dysfunctions. Degeneration in SCA3 patient brains is often seen in the dentate neurons of the cerebellum, pontine nuclei, brainstem, and basal ganglia [103,104,105]. As with most SCA diseases, SCA3 also includes a number of non-motor symptoms—including sleep and mood disorders, depression, as well as cognitive and other psychiatric disturbances [106,107].

## 7. Spinocerebellar Ataxia Type 6 (SCA6)

Spinocerebellar ataxia type 6 was first described in 1997 when researchers from the USA discovered that three different protein isoforms of the human α1A voltage-dependent calcium channel subunit (CACNA1A) contained a polymorphic CAG repeat expansion towards the C-terminal of the protein (Figure 3) [6,108,109]. SCA6 is one of only two polyglutamine SCAs (the other being SCA17) where the causative gene for the disease is not an ‘ATXN’ gene. It is also the only SCA which is not fatal, most likely due to the relatively small CAG expansion observed (Table 1) [110]. Non-CAG missense mutations found in the *CACNA1A* gene give rise to two other distinct diseases—episodic ataxia type 2 and familial hemiplegic migraine-1 [111,112,113]. Interestingly, although these autosomal dominant mutations occur in a single gene (*CACNA1A*), the three different diseases present with highly variable phenotypes. Mutations that cause SCA6 are typically CAG expansions, while episodic ataxia type 2 is associated with a loss-of-function missense mutation and familial hemiplegic migraine-1 is a gain-of-function missense mutation. A recent in-depth review of various non-CAG *CACNA1A* mutations and their associated diseases has been published by Indelicato and Boesch (2021) [114].

In regard to SCA6, the CAG expansion is located in the terminal exon (exon 47) of the *CACNA1A* gene (ENST00000360228.11) localised to 19p13. SCA6 is characterised by a relatively small expansion with a CAG-repeat range of 4–18 and a disease range of only 21–30 repeats (Figure 3) [115]. Presumably due to the limited number of repeats, the polymorphic CAGs in *CACNA1A* appear to be more stable than other CAG expansion genes [116]. Unlike most SCAs, it has been suggested that SCA6 disease severity is age dependent rather than expansion size dependent. This is not surprising since the vast majority of SCA6 patients have a pathogenic repeat of 22–24 repeats (Table 1) [115]. CACNA1A mediates the entry of calcium ions into excitable cells, with the alpha-1A isoforms giving rise to the P and Q-type calcium channels that are known as the ‘high-voltage active’ channels and thus are typically expressed in neuronal tissue [117,118,119,120].

The relatively small expansion size in SCA6 patients results in the age of onset in the sixth decade and SCA6 is typically the slowest progressing of all the SCAs [1]. This milder subtype of SCA presents with pure cerebellar ataxia and is sometimes associated with various ocular disturbances, the most common being nystagmus (repetitive, uncontrolled eye movement). The milder phenotype of SCA6 could potentially be attributed to reports that polyQ does not alter the function of CACNA1A, as mice with varying CAG lengths displayed similar fluctuations in current density [35]. However, this area is a point of contention, as historical studies suggest that CAG length directly alters the P/Q channel [121,122,123].

## 8. Spinocerebellar Ataxia Type 7 (SCA7)

Spinocerebellar ataxia type 7 was first described in 1997 and due to the severe retina phenotype, SCA7 was classified as a separate entity to the autosomal dominantly inherited ataxias [124]. Some 60 years after being first described as an ocular condition, SCA7 was finally mapped to the *ATXN7*, localised at 3p21 by three independent groups in 1995/1996 [125,126,127]. This gene was subsequently cloned and characterised, and the CAG expansion was confirmed in 1997 [128]. SCA7 is considered one of the rarest SCAs, with an estimated incidence of 1–2 per 500,000 individuals. With that being said, some large founder populations are observed in Scandinavia, Mexico, Zambia, and South Africa—in South Africa, it is the second most common form of the SCAs after SCA1 and is almost exclusively found in the native African population [129,130].

The *ATXN7* gene contains 13 exons, with the polyQ expansion residing in exon 3 of the major transcript (ENST00000295900.10), which happens to be the first protein-coding exon of the gene. The *ATXN7* gene encodes for an 892 aa, 98 kDa protein, ataxin-7 (Figure 3). Healthy individuals harbour 7–19 repeats, with incomplete penetrance seen in the range of 20–35 repeats, while a wide disease range of 36 to >400+ repeats has been reported (Table 1). It should be noted that the expansion size in *ATXN7* is the largest and most unstable of the polyQ causative genes [2]. The genetic anticipation of SCA7 is also one of the most aggressive seen in all SCAs. The ataxin-7 protein is a member of the transcriptional coactivator STAGA complex, a required coactivator for transcription of a subset of RNA polymerase II-dependent transcribed genes [131]. Ataxin-7 is predominantly localised to the nucleus in the brain and retina, and it is this nuclear localisation that is necessary to its function. It is not surprising then that the retinal phenotype observed in SCA7 is due to the conformational changes and gain-of-function of expanded ataxin-7 in photoreceptor cell nuclei of the retina [132,133,134]. Spinocerebellar ataxia type 7 is somewhat unique among SCAs by virtue of the severe retinal degeneration, known as rod-cone dystrophy, that ultimately leads to blindness [125,133]. Several in vivo studies have shown that a polyQ expansion or loss of expression of ataxin-7 causes severe dysfunction in the retina, suggesting ataxin-7 plays an essential role in retina homeostasis [133,134]. More specifically, ataxin-7 is thought to play an integral part in the interaction between the STAGA complex and rod-cone homeobox (CRX) [133,135].

As previously stated, SCA7 is caused by a highly unstable polymorphic CAG repeat in *ATXN7*, and the huge variation in CAG repeat length means that the age of onset of the disease is highly variable, occurring from infancy up to 60 years of age where infants have widespread pathogenesis that extends past typical CNS pathology. The main distinction between SCA7 and the other SCAs is the presence of the described retinal pathology, including other common characteristics such as ataxia and behavioural impairment. Recently, a newly generated SCA7 knock-in mouse model—SCA7^140Q/5Q^—represents the first knock-in *ATXN7* model that recapitulates the SCA7 phenotype, a function previous models failed to achieve [136]. Additionally, SCA7^140Q/5Q^ mice exhibited a shared disease signature to that of SCA1 and SCA2 [136], with severe clinical phenotypes observed in the cerebellum, including cerebral atrophy, peripheral nerve pathology, and photoreceptor dystrophy. This advancement provides hope that better models may lead to the discovery of a viable treatment for SCA7.

## 9. Spinocerebellar Ataxia Type 17 (SCA17)

Spinocerebellar ataxia type 17 is the most recently described polyQ SCA—Koide et al. (1999) [137] first described the disease in a 14-year-old Japanese patient who presented with unique neurological symptoms and a de novo CAG/CAA expansion mutation in *TBP* (TATA-box-binding protein). The SCA17 mutation differs from other pure CAG expansions, as it is interrupted by CAA repeats which appear to stabilise repeats so that genetic anticipation for SCA17 is far less pronounced than other SCAs [138]. Much similar to SCA6, the pathogenic repeat range for the *TBP* is relatively narrow (for most cases), and perhaps this could be attributed to the fact that SCA6 and 17 are the only two SCAs in which the gene is not an *ATXN* gene.

The *TBP* gene is the most well characterised of all SCA genes. The *TBP* gene is found at 6q27 and encodes a vital transcription initiation factor, a key component of the TFIIID complex [139,140,141]. The polymorphic CAA/CAG tract is located in exon 3 of the main *TBP* transcript, which consists of a total of 8 exons (Figure 3) [142,143]. The 37kDa TATA-Box-binding protein consists of 339 aa and is the smallest of the SCA disease-causing proteins (ENST00000230354.10). Healthy individuals have a repeat range of 25–42, incomplete penetrance is observed in individuals with 43–46 repeats, and patients suffering from SCA17 carry repeats range of 47–66 (Table 1) [128,144], although there are a few reported cases in which the repeat length was larger than 66. These lengths will typically result in a juvenile-onset form and present with a more severe phenotype and different symptoms, such as intellectual disability, muscle weakness, growth delay, and early death. However, the age of onset is in the fourth decade for the majority of cases and has a typical life span of 10–15 years following diagnosis [145].

SCA17 presents clinically with typical ataxia symptoms; however, it can be distinguished by the occurrence of frequent seizures and a high prevalence of psychiatric anomalies [144]. The predominant pathology observed in SCA17 patients, post-mortem, is significant cerebellar atrophy, which is attributed to the severe loss of Purkinje cells [144,146]. A 2020 study generated a SCA17 transgenic mouse model harbouring 109 CAG repeats in the human *TBP* transcript under the Purkinje cell-specific L7/pcp2 promoter [147]. The group aimed to investigate the neuroimaging spectrum at various symptomatic stages (pre-, early, and late stages) using magnetic resonance imaging. Although the mice displayed a normal appearance at birth, there was rapid degeneration and damage at the pre-symptomatic stage. Atrophy of the cerebellum, enlargement of the fourth ventricle, and reduced cerebellar N-acetylaspartate levels were observed at the pre-symptomatic stage. Degeneration continued as the mice progressed to the late stage, during which widespread atrophy occurred in body weight, cerebral size, and striatal size. The authors believe this particular disease model recapitulates the imaging phenotype observed in humans, providing further evidence that significant brain phenotypes occur prior to any symptomatic signs in most neurodegenerative diseases [148,149,150]. This highlights the need for a somewhat prophylactic approach to therapeutics targeting SCA diseases. Moreover, in the cases where family history is available, perhaps therapeutics may need to start years or decades prior to any physical manifestations of the disease. 

## 10. Brief Background into Antisense Therapeutics

Antisense oligonucleotides (AOs) are short (typically 18–30 bases in length) synthetic nucleic acid analogues that are designed to be complementary to their target sequence. The first report of AO-mediated gene suppression was in 1978, when Stephenson and Zamecnik showed in vitro inhibition of viral replication through what they believed to be translational arrest [151,152]. However, it was later determined that the viral repression was due to the AO-mediated cleavage and degradation of the mRNA through RNaseH induction. Although promising, AO technology was in its infancy, and this means that early unmodified or minimally modified antisense compounds were not viable therapeutic options [153,154]. Significant advances in oligonucleotide chemistries, modifications, and technologies have drastically shifted this train of thought [155,156,157,158]. Since then, great advances have been made in AO chemistries, design, and synthesis, giving rise to new mechanistic actions of these compounds. Antisense oligonucleotides now have the ability to sterically block transcription factors, suppress translation of an mRNA via redirection of the ribosome (translational blockade), alter pre-mRNA splicing, redirect polyadenylation, and even increase protein translation [159,160,161].

**Figure 4:**
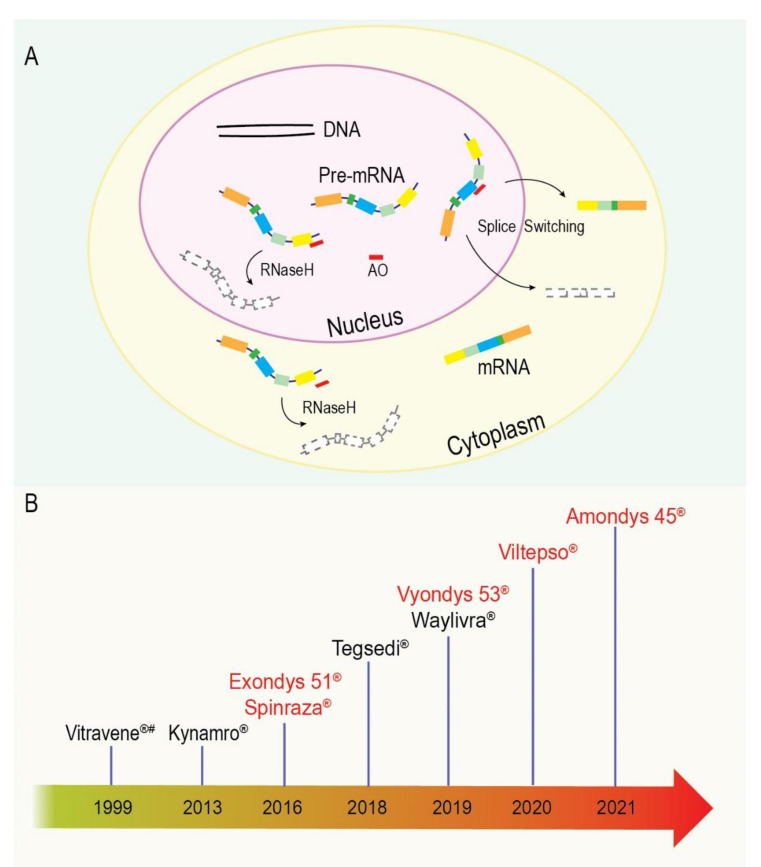
Two common antisense oligonucleotide (AO) mechanisms and the timeline of US Food and Drug Administration approvals for various AOs: (**A**) schematic of the RNaseH and splice-switching AO mechanisms. RNaseH-inducing AO results in mRNA cleavage and downregulation. Splice-switching AO induces exon skipping and/or downregulation of the target through non-sense-mediated decay. AO is depicted in red; (**B**) approved splice-switching AOs are in red, and RNaseH inducing AO are in black. # = currently withdrawn approval. Kynamro has a boxed warning ‘black box warning’. ® = registered trademark.

The activity of an AO is largely determined by the specific target site and the respective chemistry or modification of the AO. Many of these modifications were originally designed to improve the efficiency and consistency of oligonucleotide synthesis, increase resistance to nuclease degradation, and increase binding affinity or reduce toxicity [156,162,163]. Over time, and through continuous experimentation and refinement, AOs have moved beyond a simple laboratory tool and progressed into the clinic as therapeutics for serious diseases that were previously considered untreatable [164]. This advancement and procession are highlighted by the ever-growing list of approved AOs by the US Food and Drug Administration (FDA) (Figure 4). We will briefly touch on two common AO mechanisms: splice-switching through steric blocking and RNaseH-mediated degradation of a targeted mRNA (Figure 4); nevertheless, recent comprehensive reviews of chemistries, mechanistic actions, pharmacokinetics, and delivery can be found [159,165].

### 10.1. RNaseH Degradation of a Targeted RNA Transcript

RNaseH-mediated degradation of mRNA is possibly the most widely exploited of the AO-mediated mechanisms to alter gene expression. The RNaseH enzymes cleave phosphodiester bonds of RNA in a double-stranded RNA–DNA hybrid, subsequently leaving a 5′ phosphate and 3′ hydroxyl group on either end of the cleavage site [101]. There are two distinct types of RNaseH: RNaseH1 and RNaseH2 that typically have different substrate preferences. RNaseH1 is known to specifically degrade the RNA of RNA–DNA hybrid and actively engages in RNA Pol II transcription termination by degrading the R-loop RNA–DNA hybrid formation [101]. While on the other hand, RNaseH2 endonucleolytically cleaves ribonucleotides and is known to be the major source of ribonuclease H activity in mammalian cells. The RNaseH2 is also predicted to remove Okazaki fragment RNA primers during lagging strand DNA synthesis, as well as excising single ribonucleotides from DNA–DNA duplexes.

Additionally, RNaseH was the first known mechanistic action of AOs and is typically used to degrade and downregulate the target transcript. These chemistry modifications include a 2′-*O*-Methyl (2′-Me) and 2′-*O*-methoxyethyl (2′-MOE) on phosphorothioate (PS) backbones or locked nucleic acids (LNAs) ‘gapmers’—a hybrid chemistry AO that consists of wings of modified bases, typically with either 2′-Me or 2′-MOE nucleotides, flanking a DNA core—all recently thoroughly reviewed in [165,166].

### 10.2. Splice Switching

As the name suggests, splice-switching AOs can modulate the splicing of their pre-mRNA targets, achieved through the selective targeting of various motifs involved in pre-mRNA splicing. Targeting exon splicing enhancer domains inhibits targeted exon selection/recognition and leads to exon skipping [106], while AOs targeting silencer motifs that otherwise typically mediate sequence exclusion from the mature mRNA can enhance and promote the retention of selected sequences [107].

Some of the common splice-switching AOs used to date include peptide nucleic acids (PNA), 2′-Me-PS AOs, 2′-MOE-PS AOs, and phosphorodiamidate morpholino oligomers (PMOs), among others (tcDNAs and stereopure compounds)—all recently thoroughly reviewed in [165,166]. The PMOs have an excellent safety profile to date and have not shown the off-target/non-antisense effects associated with PS AOs, supporting the clinical application of PMOs, including long-term treatment [108,109,110]. Interestingly, PMOs were initially designed for translational blockade of viral replication due to their particularly high binding affinity, although no PMO-based anti-viral has yet been approved. However, our laboratory first recognised the applicability of these oligomers to splice switching in the mid-2000s [167,168,169], and subsequently, a collaboration was established to explore PMOs as agents to treat Duchenne muscular dystrophy (DMD) in association with AVI Pty Ltd, now known as Sarepta Therapeutics, Cambridge, MA. Our laboratory has since evaluated PMOs for translation blockade, exon skipping and exon inclusion, and selection of transcription start sites [170,171,172,173]. The first of our splice-switching applications to gain clinical approval is the PMO Exondys 51, targeting exon 51 of the *DMD* transcript. To date, only five commercial splice-switching AOs have been approved for therapeutic use—Spinraza (a 2′-MOE), Exondys 51, Vyondys 53, Amondys 45, and Viltepso (all PMOs) (Figure 4) [174].

## 11. Antisense Therapeutics for SCAs

Over the past two decades, there have been numerous AO-based approaches to address the expanded repeats causing the various SCAs. Here, we will focus on the most recent findings regarding potential AO therapeutics. Although siRNA- and RNAi-based therapeutics can be classified as antisense technologies, this review will only focus on splice switching and RNaseH mechanisms in relation to the causative genes of SCAs. Various RNA-silencing and gene-therapy approaches have been recently reviewed [175].

### 11.1. SCA1

As discussed in previous sections, the toxic gain-of-function of mutant *ATXN1* contributes to the SCA1 pathophysiology, thus downregulating the *ATXN1* transcript is believed to be a potential therapeutic strategy, as will be for all SCAs. Intracerebroventricular (ICV) injection of a non-allele specific gapmer resulted in a robust decrease of *Atxn1* mRNA and ATXN1 protein expression in cerebellum, cortex, pons, and medulla in a SCA1 mouse model—*Atxn1^154Q/2Q^* knock-in mouse model [176]. Moreover, significant improvement of motor performance on the balance beam and accelerating rotarod, as well as prolonged survival was observed 5 weeks after ICV gapmer injection in that mouse model. In addition, dysregulated transcriptional profile and abnormal neurochemicals were rescued by the ICV gapmer administration [176]. Similar to other non-allele-specific approaches, there are concerns over the safety of the implementation of SCA1 non-allele-specific treatment since *Atxn1*^−/−^ mice display behavioural abnormalities [177]. One study showed that this non-allele-specific AO treatment did not lead to unwanted effects regarding the functions of ATXN1-interacting proteins and the number of neuronal progenitor cells in the hippocampus of a transgenic mouse model [176]. This may be due to the fact that although suppression of *Atxn1* in these mice was significant, compared with controls, it was not absolute. Therefore, it may not be necessary or even desirable to completely knock out the SCA-related genes/proteins, as some gene products may be required for normal cellular and tissue homeostasis [2]. These findings provide support for the reduction of ATXN1 as a potential therapeutic strategy for SCA1. Furthermore, an allele-specific or CAG-specific approach was investigated using a 2′-Me-PS AO. The approach significantly reduced ATXN1 protein levels in the *Atxn1^154Q/2Q^* knock-in mouse model; however, only limited effects were observed in SCA1-patient-derived fibroblasts [178]. 

### 11.2. SCA2

Two complementary studies by Pulst et al. assessed the viability of an *Atxn2* knockout mouse model and reported a mild phenotype with no gross abnormalities in the CNS [179,180]. The phenotype observed was somewhat interesting, as the first study only described a marked increase in obesity and reduced fertility, while the subsequent study delved more deeply into behavioural analysis and discovered an impairment in amygdala plasticity, which, in turn, caused a reduction in fear and spatial learning. These data suggest that an AO-mediated ATXN2 knockdown approach may be well tolerated in SCA2 patients.

A 2017 study assessed the efficacy of 2′-MOE gapmers on *ATXN2*, via ICV injection into two humanised transgenic mouse models—the first model harboured an *ATXN2* gene with an expanded allele of 127 polyQ repeats, while the second model was a BAC transgenic mouse model expressing a full-length human *ATXN2*-containing 72 CAG repeats [181]. The gapmers were able to significantly reduce *ATXN2* mRNA (>75%) and protein expression for nearly 2.5 months. Phenotypically, the AO-mediated knockdown resulted in improved motor functioning, restored Purkinje cell function, and even normalised several dysregulated cerebellar proteins, including Rgs8, Pcp2, Pcp4, Homer3, Cep76, and Fam107b. From their findings, both mouse models responded positively to AO treatment, with similar phenotypic benefits being observed and significant downregulation of *ATXN2*.

### 11.3. SCA3

With SCA3 being the most prevalent SCA, it stands to reason that it is also the most well studied and investigated in terms of antisense therapeutics. The causative polyQ repeat of SCA3 is mapped to the penultimate exon of the *ATXN3* gene; therefore, both splice-switching and transcript-degradation approaches have been well tested in various in vitro and in vivo models [107,171,182,183,184]. From a splice-switching approach, we and others have shown that removal of exon 10 alone, as well as simultaneous exon 9 and 10 skipping, allows for the creation of an internally truncated, yet functional ATXN3 protein missing the toxic polyQ repeat [171,182,183]. Toonen et al. demonstrated that multiple ICV administrations of fully modified 2′-MOE AOs led to the production of an internally truncated ATXN3 protein in a humanised mouse model of SCA3. The group reported that exon-skipping efficiencies remained high for over 2.5 months following ICV injections, while simultaneously reducing insoluble ATXN3 protein levels and nuclear accumulation [182]. In 2019, we showed PMOs could offer a combination of benefits, as our compounds were able to significantly knockdown mutated and non-expanded ATXN3 protein levels while also inducing an internally truncated protein (missing exon 10), which represented the predominant isoform of ATXN3 following in vitro transfection of SCA3 fibroblasts [171]. With good evidence that exon skipping is a viable treatment option, we envision further development of this by Toonen et al. and their links to industry partner IONIS Pharmaceuticals.

Regarding ATXN3 downregulation strategies, studies by Paulson et al. have investigated various AO chemistries and other knockdown strategies (siRNA/RNAi) to downregulate gene and protein expression with promising success [184,185]. It was shown that a RNaseH-inducing gapmer AO induced sustained ATXN3 downregulation in a humanised SCA3 mouse model [184]. The downregulation led to 14-week prevention of oligomeric and nuclear accumulation of ATXN3 and rescued the motor impairment attributed to defects in Purkinje neuron firing frequency. Several other studies using siRNA and RNAi have also shown a successful and sustained reduction in ATXN3, with a recent review of them and other gene therapy approaches found in [175].

### 11.4. SCA7 

Due to the relative rareness of SCA6, SCA7, and SCA17 patients, comprehensive AO strategies for these diseases are limited. However, there has been some progress in the past 5 years regarding AO-mediated SCA7 therapeutics. 

With this in mind, La Spada et al. synthesised ~150 AO sequences composed of constrained ethyl nucleoside analogues targeting murine *Atxn7,* identifying a single AO with high potency and low toxicity for use in an aggressive early onset mouse model of SCA7 (SCA7 266Q knock-in model) [186]. The mouse model harbours a 266Q repeat on a small human *ATXN7* DNA fragment inserted into the endogenous mouse *Atxn7* locus [187]. Mice were subjected to a 50 μg dose of the AO directly into the vitreous humour of the eye. The *ATXN7*-targeting AO was able to induce dramatic reduction (>60%) in *ATXN7* expression and associated protein aggregation within the eyes of treated mice. The reduction of RNA and protein aggregation resulted in amelioration of vision loss, partial rescue of retinal histopathology, and photoreceptor gene expression defects in treated mice, suggesting the therapeutic potential of the *ATXN7* AO [186].

## 12. A History and Outcome of Recent Huntington’s Disease Clinical Trial

Although HD is not an SCA disease, it is caused by the same pathogenic mechanism of an expanded CAG repeat. Therefore, we considered it pertinent to briefly discuss the outcomes of a recent clinical trial using an RNaseH AO as a potential therapeutic for HD.

The initial development of Tominersen (previously known as IONIS-HTT_RX_ or RG6042) was conducted by IONIS Pharmaceuticals and began in 2005 with a proof of concept that AOs could reduce *HTT* transcript levels [188]. Further research and development led to the design and pre-clinical validation of RNaseH AO, Tominersen. This 20-mer 2′-MOE gapmer AO was designed to target and induce RNaseH1 degradation of the human *HTT* transcript, thereby lowering both wild-type and mutant (aggregation-prone) HTT protein [189]. In 2017, IONIS, in collaboration with Roche, completed a Phase I/IIa clinical trial of Tominersen (NCT02519036), where the randomised trial indicated treatment with the AO-decreased levels of mutant HTT transcript and protein, with a subsequent extension of the trial that was completed examining safety and tolerability (NCT03342053) of various doses. Following this trial, the GENERATION HD1 trial (the largest clinical trial into HD to date with 899 participants) commenced in an open-label Phase III clinical trial (NCT03761849) that tested two different dosing regimens: 120 mg intrathecal injection given either every 16 or 8 weeks [190]. Unfortunately, following a pre-planned review, the trial was halted in March 2021 on the basis of the committee issuing a ‘no go’ recommendation. At completion, participants had been dosed for a total of 69 weeks, with the patients receiving infusions every 8 weeks experiencing a greater decline in areas such as motor and cognitive decline than those receiving the placebo [190]. Additionally, although the participants on the 16-week regimen had slightly better outcomes than the 8-week regimen, they still observed no clinical benefit when compared with the placebo group [190].

Although this outcome is highly disappointing, the trial highlights the potential for further refinement of AO-mediated clinical trials into neurodegenerative diseases. Aspects such as allele-specific silencing may have greater benefits, as it is unknown whether downregulation of both *HTT* alleles may have contributed to poor outcomes. Moreover, a prophylactic approach may be needed for neurological therapies, as often symptomatic individuals have significant and irreversible neurodegeneration prior to the commencement of any treatments.

## 13. Conclusions and Future Potential

The polyQ SCAs represent six autosomal dominant diseases, all caused by a CAG repeat expansion in the coding region of their respective genes. Although caused by a similar genetic phenomenon, the clinical heterogeneity of the diseases means there is still much to be determined regarding genotype–phenotype correlations. Nevertheless, all SCAs present with neuronal inclusions, and thus, a common mechanistic action with respect to aggregation and protein dysfunction is apparent. Other common pathogenic mechanisms such as mitochondrial dysfunction, toxic RNA, and proteasomal and autophagy impairment exist between SCAs and, although not reviewed here, are the subject of therapeutic targeting. The therapeutic targeting of common pathogenic mechanisms means that a single therapeutic approach could have traversal implications for SCA diseases but also for other neurodegenerative diseases harbouring a similar mechanism. 

Although there are currently no AO-mediated clinical trials for any of the discussed SCAs, and a promising HD trial has been halted, there is room for better clinical trial design and implementation to follow. Therefore, it is highly plausible that the late-stage pre-clinical studies discussed could be in a clinical trial in the coming years that use the lessons learned from previous polyQ trials. The landscape of AO technology is expanding at a rapid pace, with an ever-growing list of new chemistries with higher specificity and lower toxicity. Moreover, AO therapeutics are gaining therapeutic approval at the fastest rate observed to date and taken together, provide great hope and potential that viable treatment options may be available to this devastating class of neurodegenerative diseases.

## Figures and Tables

**Table 1:** Genetics of the polyglutamine ataxias.

Disease	Causative Gene	Normal Gene Function *	PolyQ Location	Healthy Repeat Range	Pre-Mutation Repeat Range	Pathogenetic Repeat Range
**SCA1** MIM #164400	*ATXN1* MIM #601556	RNA metabolism and transcriptional repression	Exon 7	6–35	36–40	41–89
**SCA2** MIM #183090	*ATXN2* MIM #601517	Transcriptional repression and RNA metabolism	Exon 1	17–29	30–36	37–100+
**SCA3** MIM #109150	*ATXN3* MIM #607047	Deubiquitination and proteasomal protein degradation	Exon 10	7–44	45–54	55–89
**SCA6** MIM #183086	*CACNA1A* MIM #601011	Gives rise to P/Q calcium channels and neurotransmitter release from presynaptic terminals	Exon 47	4–18	19–20	21–30
**SCA7** MIM #164500	*ATXN7* MIM #607640	Mediates the interaction between the CRX and STAGA complex	Exon 3	7–19	20–35	36–400+
**SCA17** MIM #607136	*TBP* MIM #600075	General transcription factor and mediates the initiation of transcription through TFIID binding to the TAT box	Exon 3	25–42	43–46	47–66

SCA = spinocerebellar ataxia type; * = our current understanding of gene/protein function; # = OMIM number.

## References

[1] Buijsen R.A., Toonen L.J., Gardiner S.L., van Roon-Mom W.M. (2019). Genetics, Mechanisms, and Therapeutic Progress in Polyglutamine Spinocerebellar Ataxias. Neurotherapeutics.

[2] McIntosh C., Aung-Htut M., Fletcher S., Wilton S. (2017). Polyglutamine ataxias: From Clinical and Molecular Features to Current Therapeutic Strategies. J. Genet. Syndr. Gene Ther..

[3] Nakamura K., Jeong S.-Y., Uchihara T., Anno M., Nagashima K., Nagashima T., Ikeda S.-I., Tsuji S., Kanazawa I. (2001). SCA17, a novel autosomal dominant cerebellar ataxia caused by an expanded polyglutamine in TATA-binding protein. Hum. Mol. Genet..

[4] Paulson H.L., Shakkottai V.G., Clark H.B., Orr H.T. (2017). Polyglutamine spinocerebellar ataxias—From genes to potential treatments. Nat. Rev. Neurosci..

[5] Trottier Y., Lutz Y., Stevanin G., Imbert G. (1995). Polyglutamine expansion as a pathological epitope in Huntington’s disease and four dominant cerebellar ataxias. Nature.

[6] Zhuchenko O., Bailey J., Bonnen P., Ashizawa T., Stockton D.W., Amos C., Dobyns W.B., Subramony S., Zoghbi H.Y., Lee C.C. (1997). Autosomal dominant cerebellar ataxia (SCA6) associated with small polyglutamine expansions in the α1A-voltage-dependent calcium channel. Nat. Genet..

[7] Durr A. (2010). Autosomal dominant cerebellar ataxias: Polyglutamine expansions and beyond. Lancet Neurol..

[8] Bushart D.D., Murphy G.G., Shakkottai V.G. (2016). Precision medicine in spinocerebellar ataxias: Treatment based on common mechanisms of disease. Ann. Transl. Med..

[9] Du Y.-C., Ma Y., Shao Y.-R., Gan S.-R., Dong Y., Wu Z.-Y. (2020). Factors Associated with Intergenerational Instability of ATXN3 CAG Repeat and Genetic Anticipation in Chinese Patients with Spinocerebellar Ataxia Type 3. Cerebellum.

[10] Duenas A.M., Goold R., Giunti P. (2006). Molecular pathogenesis of spinocerebellar ataxias. Brain.

[11] Soong B.-W., Lu Y.-C., Choo K.-B., Lee H.-Y. (2001). Frequency analysis of autosomal dominant cerebellar ataxias in Taiwanese patients and clinical and molecular characterization of spinocerebellar ataxia type 6. Arch. Neurol..

[12] Fan H.-C., Ho L.-I., Chi C.-S., Chen S.-J., Peng G.-S., Chan T.-M., Lin S.-Z., Harn H.-J. (2014). Polyglutamine (PolyQ) diseases: Genetics to treatments. Cell Transplant..

[13] Ellerby L.M. (2020). Repeat Expansion Disorders: Mechanisms and Therapeutics.

[14] Paulson H. (2018). Repeat expansion diseases. Handbook of Clinical Neurology.

[15] Lee D.-Y., McMurray C.T. (2014). Trinucleotide expansion in disease: Why is there a length threshold?. Curr. Opin. Genet. Dev..

[16] La Spada A.R., Taylor J.P. (2010). Repeat expansion disease: Progress and puzzles in disease pathogenesis. Nat. Rev. Genet..

[17] Gatchel J.R., Zoghbi H.Y. (2005). Diseases of unstable repeat expansion: Mechanisms and common principles. Nat. Rev. Genet..

[18] Usdin K. (2008). The biological effects of simple tandem repeats: Lessons from the repeat expansion diseases. Genome Res..

[19] Orr H.T., Zoghbi H.Y. (2007). Trinucleotide repeat disorders. Annu. Rev. Neurosci..

[20] Mirkin S.M. (2007). Expandable DNA repeats and human disease. Nature.

[21] Petruska J., Hartenstine M.J., Goodman M.F. (1998). Analysis of strand slippage in DNA polymerase expansions of CAG/CTG triplet repeats associated with neurodegenerative disease. J. Biol. Chem..

[22] Levinson G., Gutman G.A. (1987). Slipped-strand mispairing: A major mechanism for DNA sequence evolution. Mol. Biol. Evol..

[23] Takeuchi T., Nagai Y. (2017). Protein misfolding and aggregation as a therapeutic target for polyglutamine diseases. Brain Sci..

[24] Raj K., Chanu S.I., Sarkar S. (2015). Protein misfolding and aggregation in neurodegenerative disorders: Focus on chaperone-mediated protein folding machinery. Int. J. Neurol. Res..

[25] Sweeney P., Park H., Baumann M., Dunlop J., Frydman J., Kopito R., McCampbell A., Leblanc G., Venkateswaran A., Nurmi A. (2017). Protein misfolding in neurodegenerative diseases: Implications and strategies. Transl. Neurodegener..

[26] Zoghbi H.Y., Orr H.T. (1999). Polyglutamine diseases: Protein cleavage and aggregation. Curr. Opin. Neurobiol..

[27] Ross O.A., Rutherford N.J., Baker M., Soto-Ortolaza A.I., Carrasquillo M.M., DeJesus-Hernandez M., Adamson J., Li M., Volkening K., Finger E. (2011). Ataxin-2 repeat-length variation and neurodegeneration. Hum. Mol. Genet..

[28] Soto C. (2003). Unfolding the role of protein misfolding in neurodegenerative diseases. Nat. Rev. Neurosci..

[29] Kuiper E., de Mattos E.P., Jardim L.B., Kampinga H.H., Bergink S. (2017). Chaperones in polyglutamine aggregation: Beyond the Q-stretch. Front. Neurosci..

[30] Nath S.R., Lieberman A.P. (2017). The Ubiquitination, Disaggregation and Proteasomal Degradation Machineries in Polyglutamine Disease. Front. Mol. Neurosci..

[31] Seidel K., Siswanto S., Fredrich M., Bouzrou M., Brunt E., Leeuwen F., Kampinga H., Korf H.W., Rüb U., Dunnen W. (2015). Polyglutamine aggregation in Huntington’s disease and spinocerebellar ataxia type 3: Similar mechanisms in aggregate formation. Neuropathol. Appl. Neurobiol..

[32] Burright E.N., Clark H.B., Servadio A., Matilla T., Feddersen R.M., Yunis W.S., Duvick L.A., Zoghbi H.Y., Orr H.T. (1995). SCA1 transgenic mice: A model for neurodegeneration caused by an expanded CAG trinucleotide repeat. Cell.

[33] Meierhofer D., Halbach M., Şen N.E., Gispert S., Auburger G. (2016). Ataxin-2 (Atxn2)-knock-out mice show branched chain amino acids and fatty acids pathway alterations. Mol. Cell. Proteom..

[34] Bichelmeier U., Schmidt T., Hübener J., Boy J., Rüttiger L., Häbig K., Poths S., Bonin M., Knipper M., Schmidt W.J. (2007). Nuclear localization of ataxin-3 is required for the manifestation of symptoms in SCA3: In vivo evidence. J. Neurosci..

[35] Watase K. (2015). Spinocerebellar ataxia type 6: Lessons from faithful knock-in mouse models. Neurol. Clin. Neurosci..

[36] Watase K., Barrett C.F., Miyazaki T., Ishiguro T., Ishikawa K., Hu Y., Unno T., Sun Y., Kasai S., Watanabe M. (2008). Spinocerebellar ataxia type 6 knockin mice develop a progressive neuronal dysfunction with age-dependent accumulation of mutant CaV2. 1 channels. Proc. Natl. Acad. Sci. USA.

[37] Breuer P., Haacke A., Evert B.O., Wüllner U. (2010). Nuclear aggregation of polyglutamine-expanded Ataxin-3 fragments escape the cytoplasmic quality control. J. Biol. Chem..

[38] Ansorge O., Giunti P., Michalik A., Van Broeckhoven C., Harding B., Wood N., Scaravilli F. (2004). Ataxin-7 aggregation and ubiquitination in infantile SCA7 with 180 CAG repeats. Ann. Neurol..

[39] Cornelius N., Wardman J.H., Hargreaves I.P., Neergheen V., Bie A.S., Tümer Z., Nielsen J.E., Nielsen T.T. (2017). Evidence of oxidative stress and mitochondrial dysfunction in spinocerebellar ataxia type 2 (SCA2) patient fibroblasts: Effect of coenzyme Q10 supplementation on these parameters. Mitochondrion.

[40] Hsu J.-Y., Jhang Y.-L., Cheng P.-H., Chang Y.-F., Mao S.-H., Yang H.-I., Lin C.-W., Chen C.-M., Yang S.-H. (2017). The truncated C-terminal fragment of mutant ATXN3 disrupts mitochondria dynamics in spinocerebellar ataxia type 3 models. Front. Mol. Neurosci..

[41] Laço M.N., Oliveira C.R., Paulson H.L., Rego A.C. (2012). Compromised mitochondrial complex II in models of Machado–Joseph disease. BBA Mol. Basis Dis..

[42] Furuta N., Tsukagoshi S., Hirayanagi K., Ikeda Y. (2019). Suppression of the yeast elongation factor Spt4 ortholog reduces expanded SCA36 GGCCUG repeat aggregation and cytotoxicity. Brain Res..

[43] Ito H., Fujita K., Tagawa K., Chen X., Homma H., Sasabe T., Shimizu J., Shimizu S., Tamura T., Muramatsu S.I. (2015). HMGB 1 facilitates repair of mitochondrial DNA damage and extends the lifespan of mutant ataxin-1 knock-in mice. EMBO Mol. Med..

[44] Kazachkova N., Raposo M., Montiel R., Cymbron T., Bettencourt C., Silva-Fernandes A., Silva S., Maciel P., Lima M. (2013). Patterns of mitochondrial DNA damage in blood and brain tissues of a transgenic mouse model of Machado-Joseph disease. Neurodegener. Dis..

[45] Ripolone M., Lucchini V., Ronchi D., Fagiolari G., Bordoni A., Fortunato F., Mondello S., Bonato S., Meregalli M., Torrente Y. (2018). Purkinje cell COX deficiency and mtDNA depletion in an animal model of spinocerebellar ataxia type 1. J. Neurosci. Res..

[46] Bettencourt C., Hensman-Moss D., Flower M., Wiethoff S., Brice A., Goizet C., Stevanin G., Koutsis G., Karadima G., Panas M. (2016). DNA repair pathways underlie a common genetic mechanism modulating onset in polyglutamine diseases. Ann. Neurol..

[47] Mantle D., Hargreaves I.P. (2018). Ataxia and coenzyme Q10: An overview. Br. J. Neurosci. Nurs..

[48] Fan L., Feng Y., Chen G.-C., Qin L.-Q., Fu C.-L., Chen L.-H. (2017). Effects of coenzyme Q10 supplementation on inflammatory markers: A systematic review and meta-analysis of randomized controlled trials. Pharmacol. Res..

[49] Bates C., Baxter P., Bonney H., Bremner F., Bunn L., Perez-Tome M.C., Chung M., Cipolotti L., de Silva R., Duberley K. (2016). Management of the Ataxias towards Best Clinical Practice.

[50] Torres-Ramos Y., Montoya-Estrada A., Cisneros B., Tercero-Pérez K., León-Reyes G., Leyva-García N., Hernández-Hernández O., Magaña J.J. (2018). Oxidative stress in spinocerebellar ataxia type 7 is associated with disease severity. Cerebellum.

[51] de Assis A.M., Saute J.A.M., Longoni A., Haas C.B., Torrez V.R., Brochier A.W., Souza G.N., Furtado G.V., Gheno T.C., Russo A. (2017). Peripheral oxidative stress biomarkers in spinocerebellar ataxia type 3/Machado–Joseph disease. Front. Neurol..

[52] Jimenez-Sanchez M., Thomson F., Zavodszky E., Rubinsztein D.C. (2012). Autophagy and polyglutamine diseases. Prog. Neurobiol..

[53] Sittler A., Muriel M.P., Marinello M., Brice A., den Dunnen W., Alves S. (2018). Deregulation of autophagy in postmortem brains of Machado-Joseph disease patients. Neuropathology.

[54] Nascimento-Ferreira I., Santos-Ferreira T., Sousa-Ferreira L., Auregan G., Onofre I., Alves S., Dufour N., Gould V.F.C., Koeppen A., Déglon N. (2011). Overexpression of the autophagic beclin-1 protein clears mutant ataxin-3 and alleviates Machado–Joseph disease. Brain.

[55] Liang X.H., Jackson S., Seaman M., Brown K., Kempkes B., Hibshoosh H., Levine B. (1999). Induction of autophagy and inhibition of tumorigenesis by beclin 1. Nature.

[56] Ashkenazi A., Bento C.F., Ricketts T., Vicinanza M., Siddiqi F., Pavel M., Squitieri F., Hardenberg M.C., Imarisio S., Menzies F.M. (2017). Polyglutamine tracts regulate beclin 1-dependent autophagy. Nature.

[57] Ashkenazi A., Bento C.F., Ricketts T., Vicinanza M., Siddiqi F., Pavel M., Squitieri F., Hardenberg M.C., Imarisio S., Menzies F.M. (2017). Polyglutamine tracts regulate autophagy. Autophagy.

[58] Lee D., Lee Y.-I., Lee Y.-S., Lee S.B. (2020). The mechanisms of nuclear proteotoxicity in polyglutamine spinocerebellar ataxias. Front. Neurosci..

[59] Chen I., Chang K.-H., Chen Y.-J., Chen Y.-C., Lee-Chen G.-J., Chen C.-M. (2019). Pueraria lobata and daidzein reduce cytotoxicity by enhancing ubiquitin-proteasome system function in SCA3-iPSC-derived neurons. Oxid. Med. Cell. Longev..

[60] Chen C.-M., Chen W.-L., Hung C.-T., Lin T.-H., Lee M.-C., Chen I.-C., Lin C.-H., Chao C.-Y., Wu Y.-R., Chang K.-H. (2019). Shaoyao Gancao Tang (SG-Tang), a formulated Chinese medicine, reduces aggregation and exerts neuroprotection in spinocerebellar ataxia type 17 (SCA17) cell and mouse models. Aging.

[61] Mykowska A., Sobczak K., Wojciechowska M., Kozlowski P., Krzyzosiak W.J. (2011). CAG repeats mimic CUG repeats in the misregulation of alternative splicing. Nucleic Acids Res..

[62] Scoles D.R., Ho M.H., Dansithong W., Pflieger L.T., Petersen L.W., Thai K.K., Pulst S.M. (2015). Repeat associated non-AUG translation (RAN translation) dependent on sequence downstream of the ATXN2 CAG repeat. PLoS ONE.

[63] Martí E. (2016). RNA toxicity induced by expanded CAG repeats in H untington’s disease. Brain Pathol..

[64] De Mezer M., Wojciechowska M., Napierala M., Sobczak K., Krzyzosiak W.J. (2011). Mutant CAG repeats of Huntingtin transcript fold into hairpins, form nuclear foci and are targets for RNA interference. Nucleic Acids Res..

[65] Jasinska A., Michlewski G., De Mezer M., Sobczak K., Kozlowski P., Napierala M., Krzyzosiak W.J. (2003). Structures of trinucleotide repeats in human transcripts and their functional implications. Nucleic Acids Res..

[66] Li L.B., Yu Z., Teng X., Bonini N.M. (2008). RNA toxicity is a component of ataxin-3 degeneration in Drosophila. Nature.

[67] Bhambri A., Pinto A., Pillai B. (2020). Interferon mediated neuroinflammation in polyglutamine disease is not caused by RNA toxicity. Cell Death Dis..

[68] Chiu Y.-J., Lin S.-A., Chen W.-L., Lin T.-H., Lin C.-H., Yao C.-F., Lin W., Wu Y.-R., Chang K.-H., Lee-Chen G.-J. (2020). Pathomechanism characterization and potential therapeutics identification for SCA3 targeting neuroinflammation. Aging.

[69] Rekatsina M., Paladini A., Piroli A., Zis P., Pergolizzi J.V., Varrassi G. (2020). Pathophysiology and therapeutic perspectives of oxidative stress and neurodegenerative diseases: A narrative review. Adv. Ther..

[70] Evert B.O., Vogt I.R., Kindermann C., Ozimek L., De Vos R.A., Brunt E.R., Schmitt I., Klockgether T., Wüllner U. (2001). Inflammatory genes are upregulated in expanded ataxin-3-expressing cell lines and spinocerebellar ataxia type 3 brains. J. Neurosci..

[71] Tong X., Gui H., Jin F., Heck B.W., Lin P., Ma J., Fondell J.D., Tsai C.C. (2011). Ataxin-1 and Brother of ataxin-1 are components of the Notch signalling pathway. EMBO Rep..

[72] Tsai C.-C., Kao H.-Y., Mitzutani A., Banayo E., Rajan H., McKeown M., Evans R.M. (2004). Ataxin 1, a SCA1 neurodegenerative disorder protein, is functionally linked to the silencing mediator of retinoid and thyroid hormone receptors. Proc. Natl. Acad. Sci. USA.

[73] Mizutani A., Wang L., Rajan H., Vig P.J., Alaynick W.A., Thaler J.P., Tsai C.C. (2005). Boat, an AXH domain protein, suppresses the cytotoxicity of mutant ataxin-1. EMBO J..

[74] Bowman A.B., Lam Y.C., Jafar-Nejad P., Chen H.-K., Richman R., Samaco R.C., Fryer J.D., Kahle J.J., Orr H.T., Zoghbi H.Y. (2007). Duplication of Atxn1l suppresses SCA1 neuropathology by decreasing incorporation of polyglutamine-expanded ataxin-1 into native complexes. Nat. Genet..

[75] Keiser M.S., Geoghegan J.C., Boudreau R.L., Lennox K.A., Davidson B.L. (2013). RNAi or overexpression: Alternative therapies for Spinocerebellar Ataxia Type 1. Neurobiol. Dis..

[76] Seidel K., Siswanto S., Brunt E.R., Den Dunnen W., Korf H.-W., Rüb U. (2012). Brain pathology of spinocerebellar ataxias. Acta Neuropathol..

[77] Genís D., Matilla T., Volpini V., Rosell J., Dávalos A., Ferrer I., Molins A., Estivill X. (1995). Clinical, neuropathologic, and genetic studies of a large spinocerebellar ataxia type 1 (SCA1) kindred:(CAG) n expansion and early premonitory signs and symptoms. Neurology.

[78] Stoyas C.A., La Spada A.R. (2018). The CAG–polyglutamine repeat diseases: A clinical, molecular, genetic, and pathophysiologic nosology. Handb. Clin. Neurol..

[79] Pérez L.V., Cruz G.S., Falcón N.S., Mederos L.E.A., Batallan K.E., Labrada R.R., Herrera M.P., Mesa J.M.L., Díaz J.C.R., Rodríguez R.A. (2009). Molecular epidemiology of spinocerebellar ataxias in Cuba: Insights into SCA2 founder effect in Holguin. Neurosci. Lett..

[80] Velázquez-Pérez L., Medrano-Montero J., Rodríguez-Labrada R., Canales-Ochoa N., Alí J.C., Rodes F.J.C., Graña T.R., Oliver M.O.H., Rodríguez R.A., Barrios Y.D. (2020). Hereditary ataxias in Cuba: A Nationwide epidemiological and clinical study in 1001 patients. Cerebellum.

[81] Rodríguez-Labrada R., Martins A.C., Magaña J.J., Vazquez-Mojena Y., Medrano-Montero J., Fernandez-Ruíz J., Cisneros B., Teive H., McFarland K.N., Saraiva-Pereira M.L. (2020). Founder Effects of Spinocerebellar Ataxias in the American Continents and the Caribbean. Cerebellum.

[82] Carmo-Silva S., Nobrega C., de Almeida L.P., Cavadas C. (2017). Unraveling the Role of Ataxin-2 in Metabolism. Trends Endocrinol. Metab..

[83] Pfeffer M., Gispert S., Auburger G., Wicht H., Korf H.-W. (2017). Impact of Ataxin-2 knock out on circadian locomotor behavior and PER immunoreaction in the SCN of mice. Chronobiol. Int..

[84] Ostrowski L.A., Hall A.C., Mekhail K. (2017). Ataxin-2: From RNA control to human health and disease. Genes.

[85] Lee J., Yoo E., Lee H., Park K., Hur J.-H., Lim C. (2017). LSM12 and ME31B/DDX6 define distinct modes of posttranscriptional regulation by ATAXIN-2 protein complex in Drosophila circadian pacemaker neurons. Mol. Cell.

[86] Inagaki H., Hosoda N., Tsuiji H., Hoshino S.-I. (2020). Direct evidence that Ataxin-2 is a translational activator mediating cytoplasmic polyadenylation. J. Biol. Chem..

[87] Nezhad H.G., Franklin J.P., Alix J.J., Moll T., Pattrick M., Cooper-Knock J., Shanmugarajah P., Beauchamp N.J., Hadjivissiliou M., Paling D. (2020). Simultaneous ALS and SCA2 associated with an intermediate-length ATXN2 CAG-repeat expansion. Amyotroph. Lateral Scler. Front. Degener..

[88] Elden A.C., Kim H.-J., Hart M.P., Chen-Plotkin A.S., Johnson B.S., Fang X., Armakola M., Geser F., Greene R., Lu M.M. (2010). Ataxin-2 intermediate-length polyglutamine expansions are associated with increased risk for ALS. Nature.

[89] Becker L.A., Huang B., Bieri G., Ma R., Knowles D.A., Jafar-Nejad P., Messing J., Kim H.J., Soriano A., Auburger G. (2017). Therapeutic reduction of ataxin-2 extends lifespan and reduces pathology in TDP-43 mice. Nature.

[90] Yomono H.S., Kurisaki H., Hebisawa A., Sakiyama Y., Saito Y., Murayama S. (2010). Autopsy case of SCA2 with Parkinsonian phenotype. Rinsho Shinkeigaku Clin. Neurol..

[91] Socal M., Emmel V., Rieder C., Hilbig A., Saraiva-Pereira M., Jardim L. (2009). Intrafamilial variability of Parkinson phenotype in SCAs: Novel cases due to SCA2 and SCA3 expansions. Parkinsonism Relat. Disord..

[92] Furtado S., Payami H., Lockhart P.J., Hanson M., Nutt J.G., Singleton A.A., Singleton A., Bower J., Utti R.J., Bird T.D. (2004). Profile of families with parkinsonism-predominant spinocerebellar ataxia type 2 (SCA2). Mov. Disord..

[93] Ying S., Choi S., Lee M., Perlman S., Baloh R., Toga A., Zee D. (2005). Relative atrophy of the flocculus and ocular motor dysfunction in SCA2 and SCA6. Ann. N. Y. Acad. Sci..

[94] Sasaki H., Fukazawa T., Wakisaka A., Hamada K., Hamada T., Koyama T., Tsuji S., Tashiro K. (1996). Central phenotype and related varieties of spinocerebellar ataxia 2 (SCA2): A clinical and genetic study with a pedigree in the Japanese. J. Neurol. Sci..

[95] Tang B., Liu C., Shen L., Dai H., Pan Q., Jing L., Ouyang S., Xia J. (2000). Frequency of SCA1, SCA2, SCA3/MJD, SCA6, SCA7, and DRPLA CAG trinucleotide repeat expansion in patients with hereditary spinocerebellar ataxia from Chinese kindreds. Arch. Neurol..

[96] Sudarsky L., Coutinho P. (1995). Machado-Joseph disease. Clin. Neurosci..

[97] Nakano K.K., Dawson D.M., Spence A. (1972). Machado disease A hereditary ataxia in Portuguese emigrants to Massachusetts. Neurology.

[98] Evers M.M., Toonen L.J., van Roon-Mom W.M. (2014). Ataxin-3 protein and RNA toxicity in spinocerebellar ataxia type 3: Current insights and emerging therapeutic strategies. Mol. Neurobiol..

[99] Harris G.M., Dodelzon K., Gong L., Gonzalez-Alegre P., Paulson H.L. (2010). Splice isoforms of the polyglutamine disease protein ataxin-3 exhibit similar enzymatic yet different aggregation properties. PLoS ONE.

[100] Bettencourt C., Santos C., Montiel R., do Carmo Costa M., Cruz-Morales P., Santos L.R., Simões N., Kay T., Vasconcelos J., Maciel P. (2010). Increased transcript diversity: Novel splicing variants of Machado–Joseph Disease gene (ATXN3). Neurogenetics.

[101] Bettencourt C., Santos C., Montiel R., Kay T., Vasconcelos J., Maciel P., Lima M. (2010). The (CAG) n tract of Machado–Joseph Disease gene (ATXN3): A comparison between DNA and mRNA in patients and controls. Eur. J. Hum. Genet..

[102] Ashizawa T., Öz G., Paulson H.L. (2018). Spinocerebellar ataxias: Prospects and challenges for therapy development. Nat. Rev. Neurol..

[103] Schmidt T., Landwehrmeyer G.B., Schmitt I., Trottier Y., Auburger G., Laccone F., Klockgether T., Völpel M., Epplen J.T., Schöls L. (1998). An isoform of ataxin—3 accumulates in the nucleus of neuronal cells in affected brain regions of SCA3 patients. Brain Pathol..

[104] Faber J., Schaprian T., Berkan K., Reetz K., França M.C., de Rezende T.J.R., Hong J., Liao W., van de Warrenburg B., van Gaalen J. (2021). Regional Brain and Spinal Cord Volume Loss in Spinocerebellar Ataxia Type 3. Mov. Disord..

[105] Schmidt J., Mayer A.K., Bakula D., Freude J., Weber J.J., Weiss A., Riess O., Schmidt T. (2019). Vulnerability of frontal brain neurons for the toxicity of expanded ataxin-3. Hum. Mol. Genet..

[106] Mendonça N., França M.C., Goncalves A.F., Januario C. (2018). Clinical features of Machado-Joseph disease. Adv. Exp. Med. Biol..

[107] McLoughlin H.S., Moore L.R., Paulson H.L. (2020). Pathogenesis of SCA3 and implications for other polyglutamine diseases. Neurobiol. Dis..

[108] Sasaki H., Kojima H., Yabe I., Tashiro K., Hamada T., Sawa H., Hiraga H., Nagashima K. (1998). Neuropathological and molecular studies of spinocerebellar ataxia type 6 (SCA6). Acta Neuropathol..

[109] Matsuyama Z., Kawakami H., Maruyama H., Maruyama H., Izumi Y., Komure O., Udaka F., Kameyama M., Nishio T., Kuroda Y. (1997). Molecular features of the CAG repeats of spinocerebellar ataxia 6 (SCA6). Hum. Mol. Genet..

[110] Du X., Gomez C.M. (2018). Spinocerebellar ataxia type 6: Molecular mechanisms and calcium channel genetics. Polyglutamine Disorders.

[111] Pradotto L., Mencarelli M., Bigoni M., Milesi A., Di Blasio A., Mauro A. (2016). Episodic ataxia and SCA6 within the same family due to the D302N CACNA1A gene mutation. J. Neurol. Sci..

[112] Bürk K., Kaiser F.J., Tennstedt S., Schöls L., Kreuz F.R., Wieland T., Strom T.M., Büttner T., Hollstein R., Braunholz D. (2014). A novel missense mutation in CACNA1A evaluated by in silico protein modeling is associated with non-episodic spinocerebellar ataxia with slow progression. Eur. J. Med. Genet..

[113] Barros J., Damásio J., Tuna A., Alves I., Silveira I., Pereira-Monteiro J., Sequeiros J., Alonso I., Sousa A., Coutinho P. (2013). Cerebellar Ataxia, Hemiplegic Migraine, and Related Phenotypes Due to a CACNA1A Missense Mutation: 12-year follow-up of a large Portuguese family. JAMA Neurol..

[114] Indelicato E., Boesch S. (2021). From Genotype to Phenotype: Expanding the Clinical Spectrum of CACNA1A Variants in the Era of Next Generation Sequencing. Front. Neurol..

[115] Wiethoff S., O’Connor E., Haridy N.A., Nethisinghe S., Wood N., Giunti P., Bettencourt C., Houlden H. (2018). Sequencing analysis of the SCA6 CAG expansion excludes an influence of repeat interruptions on disease onset. J. Neurol. Neurosurg. Psychiatry.

[116] Giunti P., Mantuano E., Frontali M., Veneziano L. (2015). Molecular mechanism of Spinocerebellar Ataxia type 6: Glutamine repeat disorder, channelopathy and transcriptional dysregulation. The multifaceted aspects of a single mutation. Front. Cell. Neurosci..

[117] Lipscombe D., Andrade A., Allen S.E. (2013). Alternative splicing: Functional diversity among voltage-gated calcium channels and behavioral consequences. BBA Biomembr..

[118] Pietrobon D. (2013). Calcium channels and migraine. BBA Biomembr..

[119] Rajakulendran S., Kaski D., Hanna M.G. (2012). Neuronal P/Q-type calcium channel dysfunction in inherited disorders of the CNS. Nat. Rev. Neurol..

[120] Pietrobon D. (2002). Calcium channels and channelopathies of the central nervous system. Mol. Neurobiol..

[121] Toru S., Murakoshi T., Ishikawa K., Saegusa H., Fujigasaki H., Uchihara T., Nagayama S., Osanai M., Mizusawa H., Tanabe T. (2000). Spinocerebellar ataxia type 6 mutation alters P-type calcium channel function. J. Biol. Chem..

[122] Kordasiewicz H.B., Thompson R.M., Clark H.B., Gomez C.M. (2006). C-termini of P/Q-type Ca2+ channel α1A subunits translocate to nuclei and promote polyglutamine-mediated toxicity. Hum. Mol. Genet..

[123] Matsuyama Z., Wakamori M., Mori Y., Kawakami H., Nakamura S., Imoto K. (1999). Direct alteration of the P/Q-type Ca2+ channel property by polyglutamine expansion in spinocerebellar ataxia 6. J. Neurosci.

[124] Froment J., Bonnet P., Colrat A. (1937). Heredo-degenerations retinienne et spino-cerebelleuse: Variantes ophtalmoscopiques et neurologiques presentees par trois generations successives. J. Med. Lyon.

[125] David G., Giunti P., Abbas N., Coullin P., Stevanin G., Horta W., Gemmill R., Weissenbach J., Wood N., Cunha S. (1996). The gene for autosomal dominant cerebellar ataxia type II is located in a 5-cM region in 3p12-p13: Genetic and physical mapping of the SCA7 locus. Am. J. Hum. Genet..

[126] Gouw L.G., Kaplan C.D., Haines J.H., Digre K.B., Rutledge S.L., Matilla A., Leppert M., Zoghbi H.Y., Ptácek L.J. (1995). Retinal degeneration characterizes a spinocerebellar ataxia mapping to chromosome 3p. Nat. Genet..

[127] Holmberg M., Johansson J., Forsgren L., Heijbel J., Sandgren O., Holmgren G. (1995). Localization of autosomal dominant cerebellar ataxia associated with retinal degeneration and anticipation to chromosome 3p12-p21. 1. Hum. Mol. Genet..

[128] David G., Abbas N., Stevanin G., Dürr A., Yvert G., Cancel G., Weber C., Imbert G., Saudou F., Antoniou E. (1997). Cloning of the SCA7 gene reveals a highly unstable CAG repeat expansion. Nat. Genet..

[129] Smith D., Bryer A., Watson L., Greenberg L. (2012). Inherited polyglutamine spinocerebellar ataxias in South Africa. S. Afr. Med. J..

[130] Watson L., Wood M., Smith D., Scholefield J., Ballo R., Kidson S., Greenberg L. (2016). Spinocerebellar ataxia type 7 in South Africa: Epidemiology, pathogenesis and therapy: The new millennium. S. Afr. Med. J..

[131] Helmlinger D., Hardy S., Sasorith S., Klein F., Robert F., Weber C., Miguet L., Potier N., Van-Dorsselaer A., Wurtz J.-M. (2004). Ataxin-7 is a subunit of GCN5 histone acetyltransferase-containing complexes. Hum. Mol. Genet..

[132] Kaytor M.D., Duvick L.A., Skinner P.J., Koob M.D., Ranum L.P., Orr H.T. (1999). Nuclear localization of the spinocerebellar ataxia type 7 protein, ataxin-7. Hum. Mol. Genet..

[133] La Spada A.R., Fu Y.-H., Sopher B.L., Libby R.T., Wang X., Li L.Y., Einum D.D., Huang J., Possin D.E., Smith A.C. (2001). Polyglutamine-expanded ataxin-7 antagonizes CRX function and induces cone-rod dystrophy in a mouse model of SCA7. Neuron.

[134] Mohan R.D., Dialynas G., Weake V.M., Liu J., Martin-Brown S., Florens L., Washburn M.P., Workman J.L., Abmayr S.M. (2014). Loss of Drosophila Ataxin-7, a SAGA subunit, reduces H2B ubiquitination and leads to neural and retinal degeneration. Genes Dev..

[135] Palhan V.B., Chen S., Peng G.-H., Tjernberg A., Gamper A.M., Fan Y., Chait B.T., La Spada A.R., Roeder R.G. (2005). Polyglutamine-expanded ataxin-7 inhibits STAGA histone acetyltransferase activity to produce retinal degeneration. Proc. Natl. Acad. Sci. USA.

[136] Niewiadomska-Cimicka A., Doussau F., Perot J.-B., Roux M.J., Keime C., Hache A., Piguet F., Novati A., Weber C., Yalcin B. (2021). SCA7 mouse cerebellar pathology reveals preferential downregulation of key Purkinje cell-identity genes and shared disease signature with SCA1 and SCA2. J. Neurosci..

[137] Koide R., Kobayashi S., Shimohata T., Ikeuchi T., Maruyama M., Saito M., Yamada M., Takahashi H., Tsuji S. (1999). A neurological disease caused by an expanded CAG trinucleotide repeat in the TATA-binding protein gene: A new polyglutamine disease?. Hum. Mol. Genet..

[138] Liu Q., Pan Y., Li X.-J., Li S. (2019). Molecular mechanisms and therapeutics for SCA17. Neurotherapeutics.

[139] Nikolov D.B., Hu S.-H., Lin J., Gasch A., Hoffmann A., Horikoshi M., Chua N.-H., Roeder R.G., Burley S.K. (1992). Crystal structure of TFIID TATA-box binding protein. Nature.

[140] Nikolov D.B., Chen H., Halay E.D., Hoffman A., Roeder R.G., Burley S.K. (1996). Crystal structure of a human TATA box-binding protein/TATA element complex. Proc. Natl. Acad. Sci. USA.

[141] Burley S., Roeder R. (1996). Biochemistry and structural biology of transcription factor IID (TFIID). Annu. Rev. Biochem..

[142] Reid S.J., Rees M.I., van Roon-Mom W.M., Jones A.L., MacDonald M.E., Sutherland G., During M.J., Faull R.L., Owen M.J., Dragunow M. (2003). Molecular investigation of TBP allele length:: A SCA17 cellular model and population study. Neurobiol. Dis..

[143] Yang S., Li X.-J., Li S. (2016). Molecular mechanisms underlying Spinocerebellar Ataxia 17 (SCA17) pathogenesis. Rare Dis..

[144] Rolfs A., Koeppen A.H., Bauer I., Bauer P., Buhlmann S., Topka H., Schöls L., Riess O. (2003). Clinical features and neuropathology of autosomal dominant spinocerebellar ataxia (SCA17). Ann. Neurol..

[145] Toyoshima Y., Takahashi H. (2018). Spinocerebellar ataxia type 17 (SCA17). Polyglutamine Disorders.

[146] Huang M., Verbeek D.S. (2019). Why do so many genetic insults lead to Purkinje Cell degeneration and spinocerebellar ataxia?. Neurosci. Lett..

[147] Chen C.-C., Yao N.-W., Lin C.-W., Su W.-S., Wu C.-T., Chang C., Hsieh-Li H.M. (2020). Neuroimaging Spectrum at Pre-, Early, and Late Symptomatic Stages of SCA17 Mice. Cerebellum.

[148] Cui Y., Yang S., Li X.J., Li S. (2017). Genetically modified rodent models of SCA17. J. Neurosci. Res..

[149] Fox N.C., Crum W.R., Scahill R.I., Stevens J.M., Janssen J.C., Rossor M.N. (2001). Imaging of onset and progression of Alzheimer’s disease with voxel-compression mapping of serial magnetic resonance images. Lancet.

[150] Koscik T.R., Sloat L., van der Plas E., Joers J.M., Deelchand D.K., Lenglet C., Öz G., Nopoulos P.C. (2020). Brainstem and striatal volume changes are detectable in under 1 year and predict motor decline in spinocerebellar ataxia type 1. Brain Commun..

[151] Stephenson M.L., Zamecnik P.C. (1978). Inhibition of Rous sarcoma viral RNA translation by a specific oligodeoxyribonucleotide. Proc. Natl. Acad. Sci. USA.

[152] Zamecnik P.C., Stephenson M.L. (1978). Inhibition of Rous sarcoma virus replication and cell transformation by a specific oligodeoxynucleotide. Proc. Natl. Acad. Sci. USA.

[153] Watts J.K., Brown R.H., Khvorova A. (2019). Nucleic Acid Therapeutics for Neurological Diseases. Neurotherapeutics.

[154] Khvorova A., Watts J.K. (2017). The chemical evolution of oligonucleotide therapies of clinical utility. Nat. Biotechnol..

[155] Zaw K., Greer K., Aung-Htut M.T., Veedu R.N., Fletcher S., Wilton S.D. (2019). Making the Inactive Active through Changes in Antisense Oligonucleotide Chemistries. Front. Genet..

[156] Bennett C.F. (2019). Therapeutic antisense oligonucleotides are coming of age. Annu. Rev. Med..

[157] Aung-Htut M.T., McIntosh C.S., Ham K.A., Pitout I.L., Flynn L.L., Greer K., Fletcher S., Wilton S.D. (2019). Systematic approach to developing splice modulating antisense oligonucleotides. Int. J. Mol. Sci..

[158] Rinaldi C., Wood M.J. (2018). Antisense oligonucleotides: The next frontier for treatment of neurological disorders. Nat. Rev. Neurol..

[159] Crooke S.T., Baker B.F., Crooke R.M., Liang X.-h. (2021). Antisense technology: An overview and prospectus. Nat. Rev. Drug Discov..

[160] Lim K.H., Han Z., Jeon H.Y., Kach J., Jing E., Weyn-Vanhentenryck S., Downs M., Corrionero A., Oh R., Scharner J. (2020). Antisense oligonucleotide modulation of non-productive alternative splicing upregulates gene expression. Nat. Commun..

[161] Parra M., Zhang W., Vu J., DeWitt M., Conboy J.G. (2020). Antisense targeting of decoy exons can reduce intron retention and increase protein expression in human erythroblasts. RNA.

[162] Singh N.N., Luo D., Singh R.N. (2018). Pre-mRNA splicing modulation by antisense oligonucleotides. Exon Skipping and Inclusion Therapies.

[163] Chan J.H., Lim S., Wong W.F. (2006). Antisense oligonucleotides: From design to therapeutic application. Clin. Exp. Pharmacol. Physiol..

[164] Pitout I., Flynn L.L., Wilton S.D., Fletcher S. (2019). Antisense-mediated splice intervention to treat human disease: The odyssey continues. F1000Research.

[165] Doxakis E. (2020). Therapeutic antisense oligonucleotides for movement disorders. Med. Res. Rev..

[166] Agrawal S. (2021). The Evolution of Antisense Oligonucleotide Chemistry—A Personal Journey. Biomedicines.

[167] Adams A.M., Harding P.L., Iversen P.L., Coleman C., Fletcher S., Wilton S.D. (2007). Antisense oligonucleotide induced exon skipping and the dystrophin gene transcript: Cocktails and chemistries. BMC Mol. Biol..

[168] McClorey G., Moulton H., Iversen P., Fletcher S., Wilton S. (2006). Antisense oligonucleotide-induced exon skipping restores dystrophin expression in vitro in a canine model of DMD. Gene Ther..

[169] Mann C.J., Honeyman K., McClorey G., Fletcher S., Wilton S.D. (2002). Improved antisense oligonucleotide induced exon skipping in the mdx mouse model of muscular dystrophy. J. Gene Med..

[170] Ham K.A., Aung-Htut M.T., Fletcher S., Wilton S.D. (2020). Nonsequential Splicing Events Alter Antisense-Mediated Exon Skipping Outcome in COL7A1. Int. J. Mol. Sci..

[171] McIntosh C.S., Aung-Htut M.T., Fletcher S., Wilton S.D. (2019). Removal of the Polyglutamine Repeat of Ataxin-3 by Redirecting pre-mRNA Processing. Int. J. Mol. Sci..

[172] Aung-Htut M.T., Comerford I., Johnsen R., Foyle K., Fletcher S., Wilton S.D. (2019). Reduction of integrin alpha 4 activity through splice modulating antisense oligonucleotides. Sci. Rep..

[173] Flynn L.L., Mitrpant C., Pitout I.L., Fletcher S., Wilton S.D. (2018). Antisense Oligonucleotide-Mediated Terminal Intron Retention of the SMN2 Transcript. Mol. Ther. Nucleic Acids.

[174] Dhillon S. (2020). Viltolarsen: First approval. Drugs.

[175] Afonso-Reis R., Afonso I.T., Nóbrega C. (2021). Current Status of Gene Therapy Research in Polyglutamine Spinocerebellar Ataxias. Int. J. Mol. Sci..

[176] Friedrich J., Kordasiewicz H.B., O’Callaghan B., Handler H.P., Wagener C., Duvick L., Swayze E.E., Rainwater O., Hofstra B., Benneyworth M. (2018). Antisense oligonucleotide–mediated ataxin-1 reduction prolongs survival in SCA1 mice and reveals disease-associated transcriptome profiles. JCI Insight.

[177] Matilla A., Roberson E.D., Banfi S., Morales J., Armstrong D.L., Burright E.N., Orr H.T., Sweatt J.D., Zoghbi H.Y., Matzuk M.M. (1998). Mice lacking ataxin-1 display learning deficits and decreased hippocampal paired-pulse facilitation. J. Neurosci..

[178] Kourkouta E., Weij R., González-Barriga A., Mulder M., Verheul R., Bosgra S., Groenendaal B., Puoliväli J., Toivanen J., van Deutekom J.C. (2019). Suppression of mutant protein expression in SCA3 and SCA1 mice using a CAG repeat-targeting antisense oligonucleotide. Mol. Ther. Nucleic Acids.

[179] Huynh D.P., Maalouf M., Silva A.J., Schweizer F.E., Pulst S.M. (2009). Dissociated fear and spatial learning in mice with deficiency of ataxin-2. PLoS ONE.

[180] Kiehl T.-R., Nechiporuk A., Figueroa K.P., Keating M.T., Huynh D.P., Pulst S.-M. (2006). Generation and characterization of Sca2 (ataxin-2) knockout mice. Biochem. Biophys. Res. Commun..

[181] Scoles D.R., Meera P., Schneider M.D., Paul S., Dansithong W., Figueroa K.P., Hung G., Rigo F., Bennett C.F., Otis T.S. (2017). Antisense oligonucleotide therapy for spinocerebellar ataxia type 2. Nature.

[182] Toonen L.J., Rigo F., van Attikum H., van Roon-Mom W.M. (2017). Antisense oligonucleotide-mediated removal of the polyglutamine repeat in spinocerebellar ataxia type 3 mice. Mol. Ther. Nucleic Acids.

[183] Evers M.M., Tran H.-D., Zalachoras I., Pepers B.A., Meijer O.C., den Dunnen J.T., van Ommen G.-J.B., Aartsma-Rus A., van Roon-Mom W.M. (2013). Ataxin-3 protein modification as a treatment strategy for spinocerebellar ataxia type 3: Removal of the CAG containing exon. Neurobiol. Dis..

[184] McLoughlin H.S., Moore L.R., Chopra R., Komlo R., McKenzie M., Blumenstein K.G., Zhao H., Kordasiewicz H.B., Shakkottai V.G., Paulson H.L. (2018). Oligonucleotide therapy mitigates disease in spinocerebellar ataxia type 3 mice. Ann. Neurol..

[185] Rodríguez-Lebrón E., Costa M.D., Luna-Cancalon K., Peron T.M., Fischer S., Boudreau R.L., Davidson B.L., Paulson H.L. (2013). Silencing mutant ATXN3 expression resolves molecular phenotypes in SCA3 transgenic mice. Mol. Ther..

[186] Niu C., Prakash T.P., Kim A., Quach J.L., Huryn L.A., Yang Y., Lopez E., Jazayeri A., Hung G., Sopher B.L. (2018). Antisense oligonucleotides targeting mutant Ataxin-7 restore visual function in a mouse model of spinocerebellar ataxia type 7. Sci. Transl. Med..

[187] Yoo S.-Y., Pennesi M.E., Weeber E.J., Xu B., Atkinson R., Chen S., Armstrong D.L., Wu S.M., Sweatt J.D., Zoghbi H.Y. (2003). SCA7 knockin mice model human SCA7 and reveal gradual accumulation of mutant ataxin-7 in neurons and abnormalities in short-term plasticity. Neuron.

[188] Schobel S., Palermo G., Trundell D., Kremer T., Sanwald-Ducray P., Smith A., Boak L., Doody R. A Global Development Program Testing RG6042, an Antisense Oligonucleotide, for the Treatment of Early Manifest Huntington’s Disease. Proceedings of the European Huntington’s Disease Network 2018 Plenary Meeting.

[189] Kordasiewicz H.B., Stanek L.M., Wancewicz E.V., Mazur C., McAlonis M.M., Pytel K.A., Artates J.W., Weiss A., Cheng S.H., Shihabuddin L.S. (2012). Sustained therapeutic reversal of Huntington’s disease by transient repression of huntingtin synthesis. Neuron.

[190] Kwon D. (2021). Failure of genetic therapies for Huntington’s devastates community. Nature.

